# Dynamics of Different Classes and Subclasses of Antibody Responses to Severe Acute Respiratory Syndrome Coronavirus 2 Variants after Coronavirus Disease 2019 and CoronaVac Vaccination in Thailand

**DOI:** 10.1128/msphere.00465-22

**Published:** 2023-01-23

**Authors:** Prapassorn Poolchanuan, Wasin Matsee, Sineenart Sengyee, Tanaya Siripoon, Adul Dulsuk, Rungnapa Phunpang, Phimphan Pisutsan, Watcharapong Piyaphanee, Viravarn Luvira, Narisara Chantratita

**Affiliations:** a Department of Microbiology and Immunology, Faculty of Tropical Medicine, Mahidol University, Bangkok, Thailand; b Thai Travel Clinic, Hospital for Tropical Diseases, Faculty of Tropical Medicine, Mahidol University, Bangkok, Thailand; c Department of Clinical Tropical Medicine, Faculty of Tropical Medicine, Mahidol University, Bangkok, Thailand; d Mahidol-Oxford Tropical Medicine Research Unit, Faculty of Tropical Medicine, Mahidol University, Bangkok, Thailand; University of Saskatchewan

**Keywords:** COVID-19, antibody, IgG, IgA, IgM, vaccine, SARS-CoV-2, coronavirus variants, plasma antibody, antibodies, vaccines

## Abstract

The humoral immune response plays a key role in protecting the population from SARS-CoV-2 transmission. Patients who recovered from COVID-19 as well as fully vaccinated individuals have elevated levels of antibodies. The dynamic levels of the classes and subclasses of antibody responses to new variants that occur in different populations remain unclear. We prospectively recruited 60 participants, including COVID-19 patients and CoronaVac-vaccinated individuals, in Thailand from May to August 2021. Plasma samples were collected on day 0, day 14, and day 28 to determine the dynamic levels of the classes and subclasses of plasma antibodies against the receptor-binding domain (RBD) in the spike protein (S) of four SARS-CoV-2 strains (Wuhan, Alpha, Delta, and Omicron) via enzyme-linked immunosorbent assay. Our results indicated that the patients with SARS-CoV-2 infections had broader class and subclass profiles as well as higher levels of anti-S RBD antibodies to the Wuhan, Alpha, and Delta strains than did the CoronaVac-vaccinated individuals. The median antibody levels increased and subsequently declined in a month in the COVID-19 patients and in the vaccinated group. Correlations of the classes and subclasses of antibodies were observed in the COVID-19 patients but not in the vaccinated individuals. The levels of all of the anti-S RBD antibodies against the Omicron variant were low in the patients and in the vaccinated individuals. Our study revealed distinct antibody profiles between the two cohorts, suggesting different pathways of immune activation. This could have an impact on protection from infections by new variants of concern (VOC).

**IMPORTANCE** The antibody responses to new SARS-CoV-2 variants that occur in different populations remain unclear. In this study, we recruited 60 participants, including COVID-19 patients and CoronaVac-vaccinated individuals, in Thailand and determined the dynamic levels of the IgG, IgA, IgM, and IgG subclasses of antibodies against the spike protein (S) of four SARS-CoV-2 strains. Our results showed that the patients with SARS-CoV-2 infections had broader profiles and higher levels of antibodies to the Wuhan, Alpha, and Delta strains than did the CoronaVac-vaccinated individuals. The antibody levels of both groups increased and subsequently decreased within 1 month. Higher and functional correlations of these antibodies were observed in the COVID-19 patients. The levels of all anti-S RBD antibodies against the Omicron variant were low in patients and vaccinated individuals. Our study revealed distinct antibody responses between the two groups, suggesting different pathways of immune response, which may have an impact on protection from infections by new SARS-CoV-2 variants.

## INTRODUCTION

Severe acute respiratory syndrome coronavirus 2 (SARS-CoV-2) is a causative agent of the global pandemic coronavirus disease 2019 (COVID-19), which has affected millions of people worldwide. On August 12, 2022, the World Health Organization (WHO) confirmed approximately 500 million infected cases with more than 6 million deaths (http://covid19.who.int). However, despite the administration of over 12 billion doses of vaccines to people worldwide, as reported by the WHO, COVID-19 is still increasing. Since its emergence in Wuhan, China, in late 2019, SARS-CoV-2 has evolved with multiple mutations from the original strain, and these mutations are related to significant changes in disease severity, mortality, transmissibility, and infectivity (1–3). Data by the WHO and the Global Initiative on Sharing All Influenza Data (GISAID) have shown that a series of variants of concern (VOCs) have spread and receded over time. VOCs of SARS-CoV-2 differ in spike protein (S) mutations, which potentially alter their transmissibility and pathogenicity, with the possibility of evading human immunity. Genome analyses of recent VOCs, namely, Alpha (B.1.1.7), Beta (B.1.351), Delta (B.1.617/B.1.617.2), Gamma (P.1), and Omicron (B.1.1.529), have revealed S mutations with 9 amino acids for Alpha, 18 amino acids for Beta, 9 amino acids for Delta, 17 amino acids for Gamma, and 39 amino acids for Omicron. The mutations in the receptor-binding domain (RBD) of SARS-CoV-2 S can affect its efficiency in binding to human angiotensin-converting enzyme 2 (ACE) on host cells (4–6).

The humoral immune response, a central part of the immune system, develops after vaccination and recovery from COVID-19. Many vaccines have been used globally to prevent SARS-CoV-2 infection and to reduce the severity and mortality of COVID-19, including the CoronaVac (Sinovac Biotech), BBIBP-CorV vaccine (Sinopharm), AZD1222 (Oxford-AstraZeneca), BNT162b2 (Pfizer-BioNTech), and mRNA-1273 (Moderna) vaccines. However, infections with new emerging VOCs can jeopardize vaccine efficacy in immunized individuals and patients after recovery from infections. Since its outbreak in 2019, the COVID-19 vaccine using an inactivated Wuhan-Hu 1 SARS-CoV-2 strain was first immunized in Thailand and in other Asian countries. In Thailand, two doses at 3 weeks apart have been injected into people since February of 2020 (http://ddc.moph.go.th/index.php). Subsequently, adults have received the mRNA vaccine (BNT162b2) and the mRNA-1273 or virus vector vaccine (AZD1222), the inactivated virus vaccine CoronaVac, the BBIBP-CorV vaccine (Sinopharm), or mixed vaccine boosters (http://ddc.moph.go.th/index.php). All of these vaccines were developed based on the original Wuhan strain. Reports in other countries showed that the CoronaVac vaccine provided 38% protection against SARS-CoV-2 infection, 65% against hospitalization, and 69% against severe disease outcome (7–10). In contrast, immunization with mRNA vaccines or virus vectors has shown more efficacy, but these vaccinations may not completely protect humans from VOC infections (1, 4).

When most of the population has been infected with SARS-CoV-2 and most people receive the COVID-19 vaccine, breakthrough infections can occur in some individuals. To further prevent and control emerging VOC transmission, it is important to understand the immune responses and correlated protection against diverse VOCs in patients with prior infections versus vaccinated individuals in the population. The classes and subclasses of antibody responses to many pathogens mediate distinct biological functions, such as neutralization, complement fixation, and antibody-dependent, medicated cell cytotoxicity (ADCC) (11, 12). Longitudinal studies of antibody responses after COVID-19 have revealed increased IgM, IgA, and IgG levels in the serum or plasma against S and the nucleocapsid protein (N) of SARS-CoV-2 after disease onset (13–20). In addition, vaccination with mRNA vaccines can induce different classes of antibodies against the Wuhan strain in the serum and saliva of human subjects (17, 21, 22). These studies have performed experiments using a wild-type strain (Wuhan). However, it is unclear which dynamic levels of the classes and subclasses of antibody responses to new VOCs occur in different populations that have received different vaccines, and it is also unclear which antibody profiles occur in patients after recovery from COVID-19, compared with individuals who received the CoronaVac vaccination.

Therefore, we prospectively recruited 60 participants. The sample was comprised of COVID-19 patients and vaccinated individuals who received two doses of the CoronaVac vaccine in Thailand to determine the classes and subclasses of the antibody responses to the S RBD of four SARS-CoV-2 strains (Wuhan, Alpha, Delta, and Omicron). The dynamic levels of plasma IgM, IgA, IgG, and IgG subclasses of specific antibody responses to the SARS-CoV-2 Wuhan strain and variants in COVID-19 patients and in CoronaVac-vaccinated individuals were measured at three different time points over 28 days using enzyme-linked immunosorbent assay (ELISA). We also determined neutralizing antibodies against the SARS-CoV-2 variants.

## RESULTS

### Characteristics of COVID-19 patients and vaccinated individuals.

We recruited 60 participants. The sample was comprised of 30 COVID-19 patients and 30 vaccinated individuals. The clinical characteristics of the COVID-19 patients are shown in Table 1. The median age of the COVID-19 patients was 46 years (interquartile range [IQR], 33 to 63 years) and 16 patients (53.3%) were male. 12 patients (40%) had no vaccination history before the infection. 16 (53.3%) patients had comorbidities. Of the total patients, 26.7% had hypertension, 20.0% had dyslipidemia, 13.3% had diabetes, and 13.3% had obesity (BMI > 30). The median duration of patient hospital stay was 10.5 (IQR, 9.8 to 14). 24 (80%) patients had pneumonia. 24 (80%) patients had lung involvement and complications related to a COVID-19 infection. None of the patients in this cohort died in the hospital. The median age of the vaccinated group was 42.5 years (IQR, 30 to 50 years). 9 patients (30%) were male.

**TABLE 1:** Demographics and clinical characteristics of COVID-19 patients and vaccinated individuals

Characteristics	COVID-19 patients (*n* = 30)	Vaccinated individuals (*n* = 30)^*a*^
Demographics		
Age in years, median (IQR)	46 (33 to 63)	42.5 (30 to 50)
Male; no. (%)	16 (53.3%)	9 (30%)
Female; no. (%)	14 (46.7%)	21 (70%)
Vaccination history; no. (%)		
No dose	12 (40.0%)	30 (100%)
One dose	10 (33.3%)	0
Two doses	7 (23.3%)	0
Three doses	1 (3.3%)	0
Preexisting conditions; no. (%)		
No underlying disease	14 (46.7%)	19 (63.3%)
Hypertension	8 (26.7%)	5 (16.7%)
Dyslipidemia	6 (20.0%)	4 (13.3%)
Diabetes	4 (13.3%)	2 (6.7%)
Chronic heart disease	4 (13.3%)	0
Obesity (BMI > 30)	4 (13.3%)	3 (10%)
Chronic hematologic disease	3 (10.0%)	1 (3.3%)
Chronic lung disease	1 (3.3%)	0
Asthma	1 (3.3%)	1 (3.3%)
Cancer	1 (3.3%)	0
Moderate to severe liver disease	1 (3.3%)	0
Chronic neurological disease	1 (3.3%)	0
Rheumatologic disease	1 (3.3%)	0
Duration of symptom onset, median (IQR) in days	5 (2 to 6)	ND
Days to enrollment, median (IQR)	1 (0 to 2)	ND
Duration of hospital stay, median (IQR)	10.5 (9.8 to 14)	ND
Pneumonia, N (%)	24 (80%)	ND
Organ involved and complications, N (%)		
Lung	24 (80%)	ND
Liver	10 (33.3%)	ND
Kidney	2 (6.7%)	ND
Died in hospital, N	0	ND

a ND, not determined.

To confirm no SARS-CoV-2 infection prior to the vaccination, we determined the antibody levels against the SARS-CoV-2 N protein in the plasma of the 30 vaccinated patients on day 0 (pre-vaccination) via electro-chemiluminescence immunoassay (ECLIA) assay. None of these pre-vaccinated samples were anti-N antibody positive (COI < 1.0) (Fig. S1), suggesting that they did not have previous SARS-CoV-2 infections before enrollment.

10.1128/msphere.00465-22.1 FIG S1 Anti-nucleocapsid (N) antibody to SARS-CoV-2 in the plasma of 30 prevaccinated individuals on day 0. The anti-N antibody was determined by ECLIA. The nonreactive results were a value range with a cutoff index (COI) of <1.0. Download FIG S1, DOCX file, 0.07 MB. Copyright © 2023 Poolchanuan et al. 2023 Poolchanuan et al. https://creativecommons.org/licenses/by/4.0/ This content is distributed under the terms of the Creative Commons Attribution 4.0 International license.

### Dynamic levels of IgM, IgA, and IgG antibodies against SARS-CoV-2 variants in COVID-19 patients.

**FIG 1:**
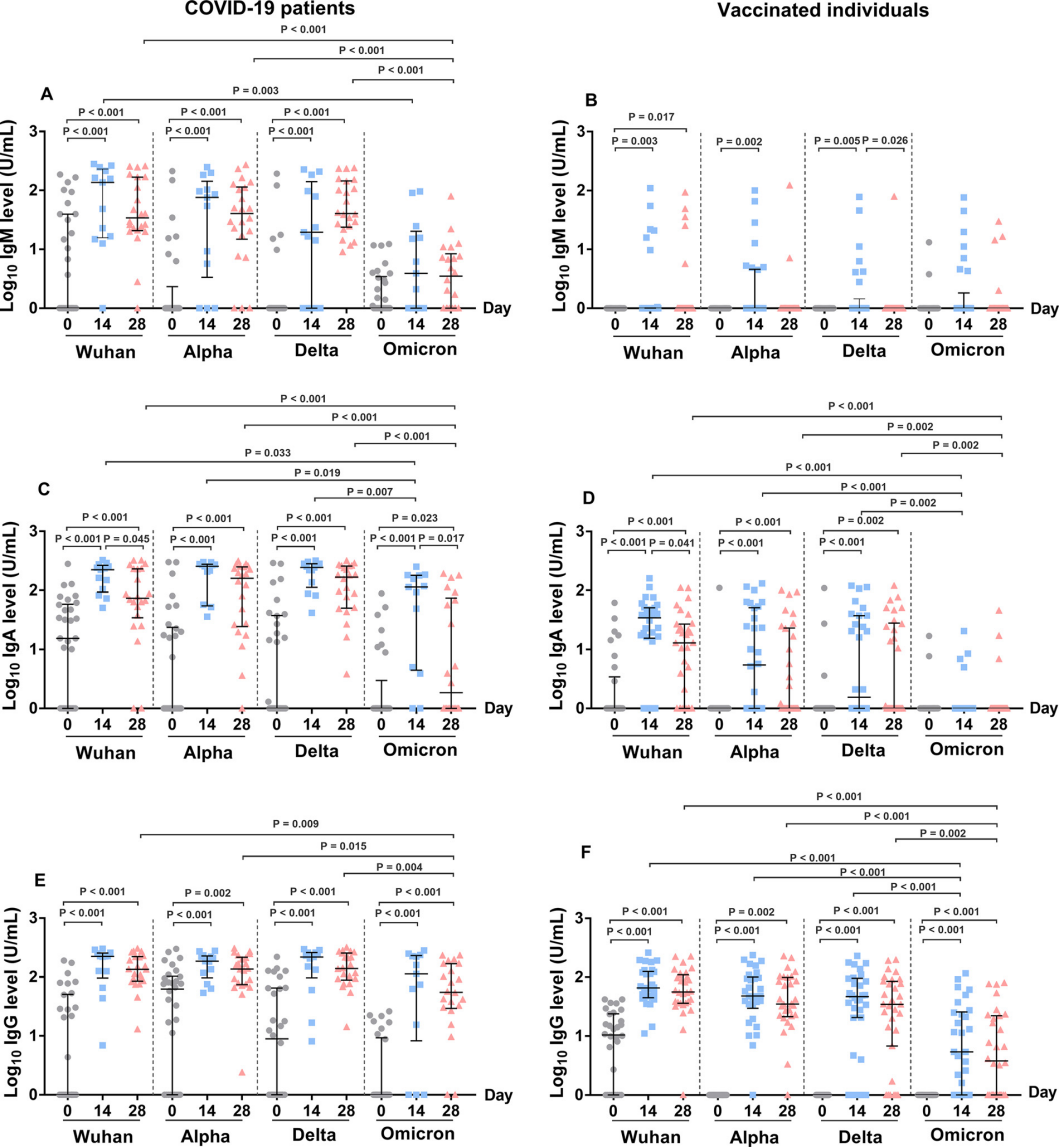
Levels of IgM, IgA, and IgG antibodies against the S RBD protein of SARS-CoV-2 variants in COVID-19 patients (A, C, and E) and in vaccinated individuals (B, D, and F) at different time points. Antibodies were determined by ELISA from the plasma samples of patients at the time of diagnosis (day 0) (*n* = 30), day 14 (*n* = 13), and day 28 (*n* = 22) as well as from the plasma samples of vaccinated individuals prior to vaccination (day 0) (*n* = 30), day 14 (*n* = 30) and day 28 (*n* = 30) after the second dose of vaccination. (A and B) IgM level, (C and D) IgA level, and (E and F) IgG level. The Mann-Whitney test was used to compare the median differences between groups.

Plasma IgM, IgA, and IgG antibody levels against the S RBD of the Wuhan strain, as well as the Alpha, Delta, and Omicron variants, were determined at three different time points, and the results were compared between COVID-19 patients and CoronaVac-vaccinated individuals (Fig. 1; Table 2).

**TABLE 2:** Median levels with IQR (U/mL) of classes and subclasses of antibody responses against the S SARS-CoV-2 Wuhan and VOCs

Antibody^*a*^	Median (IQR) of antibodies against RBD of SARS-CoV-2 strains											
	Wuhan			Alpha (B.1.1.7) variant			Delta (B.1.617.2) variant			Omicron (B.1.1.529) variant		
	Day 0^*b*^	Day 14	Day 28	Day 0	Day 14	Day 28	Day 0	Day 14	Day 28	Day 0	Day 14	Day 28
Antibody in COVID-19 patients												
IgM	0 (0 to 39.6)	136.9 (15.7 to 231.0)	34.2 (20.8 to 167.8)	0 (0 to 1.6)	76.2 (2.9 to 143.2)	40.6 (14.9 to 114.3)	0	19.5 (0 to 140.9)	40.5 (23.8 to 144.7)	0.9 (0 to 3.5)	3.9 (0 to 20.3)	3.5 (0.2 to 8.4)
IgA	15.4 (0 to 58.4)	223.4 (93.9 to 264.3)	73.8 (34.5 to 230.4)	0 (0 to 23.7)	255.4 (54.9 to 275.7)	159.7 (24.4 to 248.4)	0 (0 to 37.6)	243.0 (112.1 to 282.0)	167.3 (50.3 to 257.2)	0 (0 to 0.1)	113.7 (4.5 to 179.5)	1.4 (0 to 74.0)
IgG	0 (0 to 50.7)	223.8 (95.5 to 254.5)	135.2 (84.9 to 222.0)	62.3 (0 to 103.1)	185.1 (96.6 to 228.0)	136.9 (74.1 to 216.4)	11.3 (0 to 64.8)	218.7 (96.6 to 260.3)	139.6 (88.2 to 254.9)	0 (0 to 9.3)	113.2 (8.2 to 231.4)	55.0 (29.3 to 168.4)
IgG1	0 (0 to 12.5)	352.1 (125.9 to 361.9)	333.9 (243.4 to 349.5)	0 (0 to 10.3)	349.9 (112.5 to 364.4)	326.2 (204.0 to 343.5)	1.1 (0 to 24.7)	354.8 (246.5 to 365.5)	338.1 (278.1 to 351.6)	1.2 (0.5 to 2.3)	50.9 (1.6 to 322.4)	32.9 (2.9 to 178.9)
IgG2	11.4 (0.9 to 27.5)	17.7 (12.4 to 56.9)	26.4 (8.1 to 45.5)	8.8 (1.5 to 26.0)	14.5 (7.3 to 56.4)	18.7 (5.4 to 30.0)	10.7 (5.5 to 44.0)	17.7 (8.4 to 57.7)	13.4 (0 to 34.9)	0	0 (0 to 2.4)	0
IgG3	9.1 (0 to 36.3)	329.8 (139.0 to 336.4)	268.2 (172.0 to 289.5)	9.4 (0 to 33.9)	327.5 (126.5 to 331.4)	258.2 (162.5 to 275.4)	12.4 (0 to 28.5)	327.0 (143.9 to 341.8)	267.4 (171.0 to 315.7)	0 (0 to 2.8)	46.1 (7.9 to 170.7)	19.2 (6.0 to 38.5)
IgG4	40.0 (3.9 to 120.7)	60.6 (3.0 to 246.3)	28.6 (3.2 to 93.7)	34.8 (4.3 to 109.6)	51.3 (2.0 to 221.7)	22.6 (7.6 to 87.9)	30.8 (0.8 to 171.9)	47.5 (7.0 to 274.8)	33.3 (6.0 to 188.8)	0 (0 to 2.1)	0.7 (0 to 3.5)	0 (0 to 0.7)
Antibody in CoronaVac-vaccinated individuals												
IgM	0	0 (0 to 0.3)	0	0	0 (0 to 4.6)	0	0	0 (0 to 1.2)	0	0	(0 to 1.1)	0
IgA	0 (0 to 3.4)	34.5 (15.6 to 51.2)	13.0 (0 to 27.0)	0	5.5 (0 to 51.2)	0.2 (0 to 23.2)	0	1.1 (0 to 37.5)	0 (0 to 28.1)	0	0	0
IgG	10.5 (0 to 24.0)	65.6 (44.9 to 125.4)	56.0 (36.1 to 110.1)	0	47.9 (29.7 to 100.6)	35.0 (21.4 to 98.9)	0	46.7 (20.7 to 95.5)	34.6 (6.8 to 85.1)	0	5.4 (0 to 25.7)	3.8 (0 to 22.2)
IgG1	0	38.7 (7.5 to 148.9)	75.2 (17.8 to 143.7)	0	13.2 (0 to 68.8)	0.2 (0 to 23.7)	0.1 (0 to 2.0)	39.6 (13.9 to 125.0)	20.6 (8.9 to 54.4)	0 (0 to 0.4)	1.2 (0 to 2.4)	1.6 (0.9 to 2.5)
IgG2	0 (0 to 0.2)	0 (0 to 1.6)	0 (0 to 25.7)	0	0	0	0 (0 to 2.0)	0 (0 to 2.6)	0 (0 to 7.3)	0	0	0 (0 to 0.5)
IgG3	0 (0 to 0.5)	48.8 (17.4 to 89.2)	21.4 (4.5 to 61.4)	0	37.7 (16.4 to 86.9)	9.0 (0 to 34.2)	0.7 (0 to 16.8)	56.2 (32.3 to 103.1)	16.8 (1.4 to 44.5)	0 (0 to 0.2)	5.8 (1.3 to 25.4)	5.8 (2.5 to 16.1)
IgG4	0	0.1 (0 to 1.5)	0	0	0	0	0 (0 to 0.4)	4.6 (0 to 35.5)	21.7 (1.1 to 42.1)	0 (0 to 0.5)	0	0 (0 to 0.6)

a The antibodies were determined against RBD proteins by ELISA in 30 COVID-19 patients and in 30 vaccinated individuals at three different time points.

b The plasma samples of the patients were obtained at the time of diagnosis (day 0), on day 14, and on day 28. The plasma samples of the vaccinated individuals were obtained prior to vaccination (day 0), on day 14, and on day 28 after the second dose of vaccination.

In the COVID-19 patients, the IgM antibodies against the S RBD of the Wuhan strain and the Alpha and Delta variants significantly increased on day 14 after disease onset (compared to day 0, all *P* < 0.001) and subsequently declined on day 28 after enrollment (Fig. 1A). The median level of plasma IgM in patients did not increase for the Omicron variant on day 14 and day 28. The lack of increase in IgM against the Omicron variant after infection suggests that the COVID-19 patients in this cohort had an ineffective IgM antibody response against the S RBD of the Omicron variant. In contrast, the median levels of plasma IgA and IgG in the COVID-19 patients against the Wuhan strain and all of the SARS-CoV-2 variants increased significantly on day 14 (compared to day 0, all *P* < 0.001) and declined on day 28 (Fig. 1C and E). The levels of IgA and IgG against the Omicron variant exceeded that of IgM. However, these IgA and IgG antibodies against the Omicron variant were lower than those against the Wuhan, Alpha, and Delta variants. The data suggest that these patients had high IgA and IgG cross-reacting antibody responses among more closely related lineages of infecting strains (Wuhan or Alpha or Delta) and that the levels declined in a month. These antibodies showed low cross-reactivities with the S RBD of the Omicron variant.

18 of the 30 COVID-19 patients had vaccination histories before their SARS-CoV-2 infections. The vaccination details are provided in Table S1. We compared antibody levels between the patients who had (*n* = 18) and did not have (*n* = 12) vaccination histories. The levels of IgM and IgA at all time points were not much different between the two groups (Fig. S2A and B; Tables S2 and S3). However, the plasma of the vaccinated patients showed preexisting IgG levels against the S RBD of the Wuhan, Alpha, and Delta strains on the first day of enrollment, compared with the unvaccinated patients (all *P* < 0.05). The IgG levels on day 28 against the Omicron variant in the vaccinated COVID-19 patients were higher than those in the unvaccinated patients (*P* = 0.013) (Fig. S2C; Tables S2 and S3).

10.1128/msphere.00465-22.2 FIG S2 Levels of IgM, IgA, and IgG antibodies against the S RBD protein of SARS-CoV-2 variants in unvaccinated COVID-19 patients (*n* = 12) and in vaccinated COVID-19 patients (*n* = 18) at different time points. The antibodies were determined by ELISA from the plasma of patients at the time of diagnosis (day 0), day 14, and day 28. (A) IgM level. (B) IgA level. (C) IgG level. Download FIG S2, DOCX file, 0.4 MB. Copyright © 2023 Poolchanuan et al. 2023 Poolchanuan et al. https://creativecommons.org/licenses/by/4.0/ This content is distributed under the terms of the Creative Commons Attribution 4.0 International license.

10.1128/msphere.00465-22.8 TABLE S1 Type of vaccine and vaccination period in 30 COVID-19 patients prior to their infections. Download Table S1, DOCX file, 0.01 MB. Copyright © 2023 Poolchanuan et al. 2023 Poolchanuan et al. https://creativecommons.org/licenses/by/4.0/ This content is distributed under the terms of the Creative Commons Attribution 4.0 International license.

10.1128/msphere.00465-22.9 TABLE S2 Median levels with IQR (U/mL) of the classes and subclasses of antibody responses against the S SARS-CoV-2 Wuhan and VOCs. The antibodies were determined against RBD proteins by ELISA in 18 vaccinated COVID-19 patients at three different time points. Plasma samples of the patients were obtained at the time of diagnosis (day 0), day 14, and day 28. Download Table S2, DOCX file, 0.01 MB. Copyright © 2023 Poolchanuan et al. 2023 Poolchanuan et al. https://creativecommons.org/licenses/by/4.0/ This content is distributed under the terms of the Creative Commons Attribution 4.0 International license.

10.1128/msphere.00465-22.10 TABLE S3 Median levels with IQR (U/mL) of the classes and subclasses of antibody responses against the S SARS-CoV-2 Wuhan and VOCs. The antibodies were determined against RBD proteins by ELISA in 12 unvaccinated COVID-19 patients at three different time points. Plasma samples of the patients were obtained at the time of diagnosis (day 0), day 14, and day 28. Download Table S3, DOCX file, 0.01 MB. Copyright © 2023 Poolchanuan et al. 2023 Poolchanuan et al. https://creativecommons.org/licenses/by/4.0/ This content is distributed under the terms of the Creative Commons Attribution 4.0 International license.

The median levels of IgM, IgA, and IgG antibodies, after excluding the data of the 18 COVID-19 patients with vaccination history, are shown in Table S3. The data confirm that the COVID-19 patients had high IgA and IgG cross-reacting antibody responses among the Wuhan strain as well as the Alpha and Delta variants, but not the Omicron variant, and these antibodies showed low cross-reactivities with the S RBD of the Omicron variant. The levels of these antibodies in the patients were increased by day 14 and declined toward day 28.

### Dynamic levels of IgM, IgA, and IgG antibodies against SARS-CoV-2 variants in CoronaVac-vaccinated individuals.

Interestingly, the median levels of IgM in the CoronaVac-vaccinated individuals against all of the SARS-CoV-2 strains were low (all medians = 0), compared with those of the COVID-19 patients (Fig. 1B; Table 2). Compared with IgM, the median levels of IgA and IgG against the Wuhan strain as well as the Alpha and Delta variants significantly increased after 14 days of the second dose of CoronaVac vaccination (all *P* < 0.001) (Fig. 1D and F). Similar to the results of the COVID-19 patients, the IgA and IgG levels of CoronaVac-vaccinated individuals decreased on day 28. The IgG levels against the Wuhan strain as well as the Alpha and Delta variants exceeded the IgA levels, suggesting that IgG was the predominant antibody class in response to the CoronaVac vaccination. However, we observed only a slight increase in S RBD-specific IgG but not IgM antibodies against the Omicron variant on days 14 and 28, compared to day 0 (IgG, median [IQR], 5.4 (0 to 25.7) versus 0 for day 14 and 3.8 (0 to 22.2) versus 0 for day 28). The data suggest that the antibody responses in the individuals after the CoronaVac vaccination did not completely bind strongly to the S RBD of the Omicron variant.

We compared the median levels of IgM, IgA, and IgG antibodies between (i) unvaccinated COVID-19 patients and CoronaVac-vaccinated individuals and (ii) vaccinated COVID-19 patients and CoronaVac-vaccinated individuals. The levels of these antibodies in the COVID-19 patients, both vaccinated and unvaccinated, were higher than the levels observed in the CoronaVac-vaccinated individuals.

### Dynamic levels of IgG subclasses of antibodies against SARS-CoV-2 variants in COVID-19 patients.

**FIG 2:**
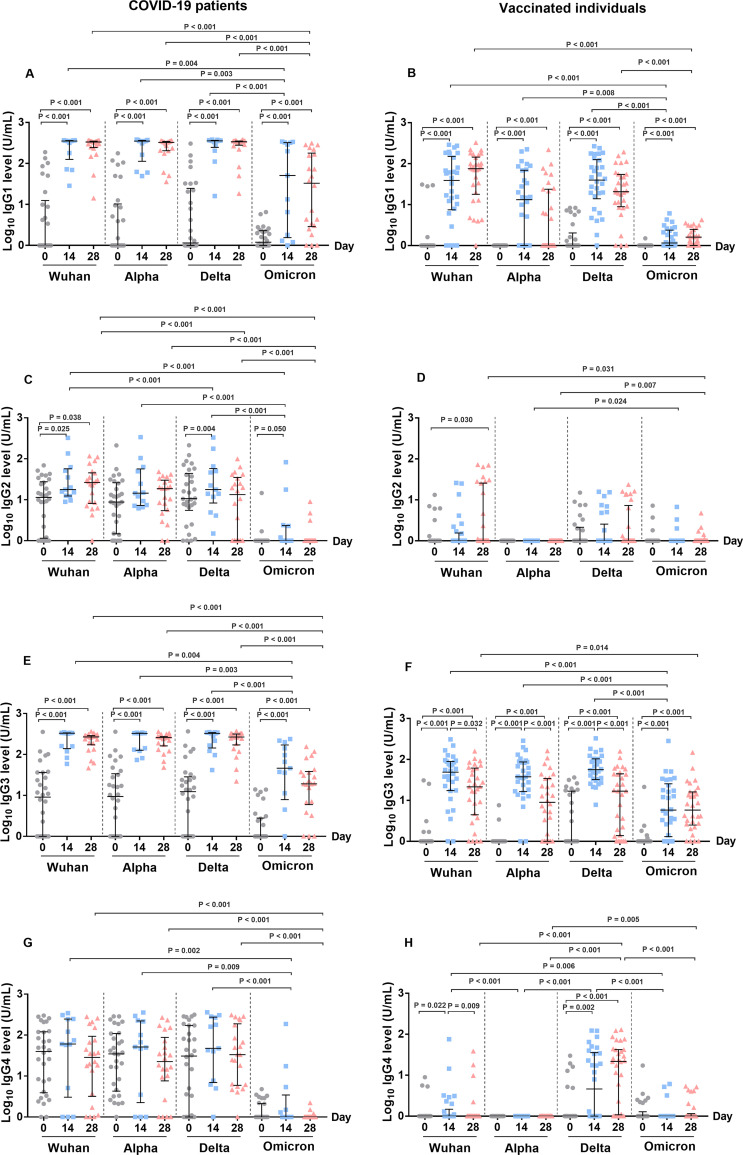
Levels of IgG subclasses of antibody response to the S RBD protein of SARS-CoV-2 variants in COVID-19 patients (A, C, E, and G) and vaccinated individuals (B, D, F, and H). Antibodies were determined from the plasma samples of patients at the time of diagnosis (day 0) (*n* = 30), day 14 (*n* = 13), and day 28 (*n* = 22) as well as from the plasma samples of vaccinated individuals prior to vaccination (day 0) (*n* = 30), day 14 (*n* = 30), and day 28 (*n* = 30) after the second dose of vaccination. (A and B) IgG1 level, (C and D) IgG2 level, (E and F) IgG3 level, and (G and H) IgG4 level. The Mann-Whitney test was used to compare the median differences between groups.

We further compared the changes in the IgG subclass responses to the SARS-CoV-2 variants in the plasma samples of COVID-19 patients and CoronaVac-vaccinated individuals (Fig. 2; Table 2).

In the COVID-19 patients, IgG1 and IgG3 were the predominant IgG subclasses in response to infection, and these were followed by IgG4 and IgG2. We found that the median IgG1 and IgG3 levels against the Wuhan strain and the VOCs (Alpha, Delta, and Omicron variants) significantly increased on day 14 after the admission of the patients (compared to day 0, all *P* < 0.001) and subsequently declined on day 28 post-enrollment (Fig. 2A and E). However, the IgG1 and IgG3 levels on day 14 and day 28 against the Omicron variant were significantly lower than those against the Wuhan, Alpha, and Delta variants. The levels of the IgG2 antibody against the Wuhan, Delta, and Omicron variants on day 0 were low and slightly increased on day 14 and day 28, though this was observed for the Wuhan strain only (Fig. 2C). The median level of the IgG4 antibody in the plasma of the COVID-19 patients against all of the variants was detectable but did not significantly change on day 14 and day 28 after enrollment (Fig. 2G). Similar to the IgG2 levels, we did not detect IgG4 levels against the S RBD of the Omicron variant.

Next, we compared the changes in the IgG subclass responses to the SARS-CoV-2 variants in the plasma samples of the COVID-19 patients who received and did not receive a vaccine prior to infection. We observed existing IgG1 and IgG3 against the Wuhan strain as well as the Alpha and Delta variants on day 0 in the vaccinated patients, compared to the unvaccinated patients (*P* < 0.05) (Fig. S3A and C). On day 28, the IgG1 levels against the Omicron variant for the vaccinated patients was significantly higher than those of the unvaccinated patients (*P* < 0.001) (Fig. S3A). The IgG2 and IgG 4 levels did not differ between the two groups (Fig. S3B and D).

10.1128/msphere.00465-22.3 FIG S3 Levels of IgG1, IgG2, IgG3, and IgG4 antibodies against the S RBD protein of SARS-CoV-2 variants in unvaccinated COVID-19 patients (*n* = 12) and in vaccinated COVID-19 patients (*n* = 18) at different time points. Antibodies were determined by ELISA from the plasma of patients at the time of diagnosis (day 0), day 14, and day 28. (A) IgG1 level. (B) IgG2 level. (C) IgG3 level. (D) IgG4 level. Download FIG S3, DOCX file, 0.2 MB. Copyright © 2023 Poolchanuan et al. 2023 Poolchanuan et al. https://creativecommons.org/licenses/by/4.0/ This content is distributed under the terms of the Creative Commons Attribution 4.0 International license.

The antibody levels, after excluding the data of the 18 vaccinated COVID-19 patients, are shown in Table S3. We observed that the unvaccinated patients showed low IgG3 responses, but we did not detect increased IgG1, IgG2, or IgG4 levels against the Omicron variant at any time point. However, the plasma of the unvaccinated patients showed the same changes for all of the IgG subclass responses to the Wuhan strain as well as to the Alpha and Delta variants on day 14 and day 28, as shown in the combined data for the unvaccinated and vaccinated patients, which is presented in Fig. 2.

### The dynamic levels of the IgG subclasses of antibodies against the SARS-CoV-2 variants in the CoronaVac-vaccinated individuals.

Meanwhile, we observed that IgG1 and IgG3 were the predominant IgG subclasses induced by the CoronaVac vaccination (Fig. 2). The S RBD-specific IgG1 and IgG3 antibodies against the Wuhan strain as well as the Alpha and Delta variants significantly increased on day 14, compared with day 0 (all *P* < 0.001), and the IgG3 levels subsequently declined on day 28 (Fig. 2B and F). However, the IgG3 level against the Omicron variant was lower than those observed against the Wuhan strain as well as the Alpha and Delta variants. The median level of IgG2 that was induced by the CoronaVac vaccination against all of the SARS-CoV-2 variants was low (all medians = 0) (Fig. 2D; Table 2). The IgG4 level against the Delta variant significantly increased on day 14 (*P* = 0.002) after the CoronaVac vaccination, but it was undetectable against the Alpha and Omicron variants (Fig. 2H).

We compared the median levels of IgG1, IgG2, IgG3, and IgG4 antibodies between (i) unvaccinated COVID-19 patients and CoronaVac-vaccinated individuals and (ii) vaccinated COVID-19 patients and CoronaVac-vaccinated individuals. The levels of these antibodies in the COVID-19 patients, both vaccinated and unvaccinated, were higher than the levels observed in the CoronaVac-vaccinated individuals. The results showed higher antibody levels in the COVID-19 patients than in the CoronaVac-vaccinated individuals for both comparisons.

### Correlation of different classes of antibodies against the S RBD of the SARS-CoV-2 variants in COVID-19 patients and in CoronaVac-vaccinated individuals.

**FIG 3:**
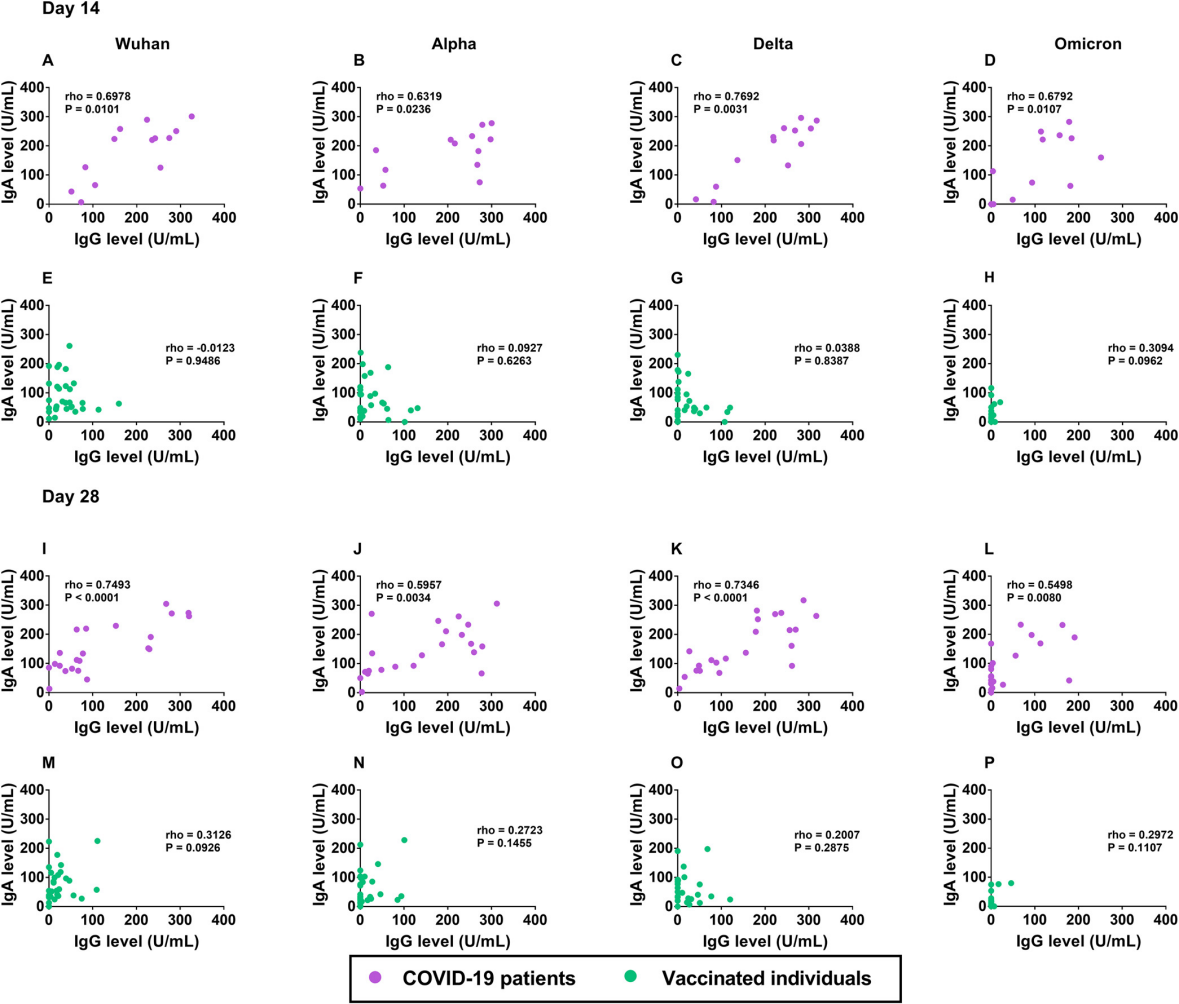
Correlation between the IgA and IgG antibody levels to S SARS-CoV-2 Wuhan and variants on day 14 (A–H) and day 28 (I–P) in COVID-19 patients and in CoronaVac-vaccinated individuals. (A, E, I, and M) IgA versus IgG antibody against the Wuhan strain. (B, F, J, and N) IgA versus IgG antibody against the Alpha variant. (C, G, K, and O) IgA versus IgG antibody against the Delta variant. (D, H, L, and P) IgA versus IgG antibody against the Omicron variant. Antibodies were determined from the plasma samples of COVID-19 patients on day 14 (*n* = 13), day 28 (*n* = 22) as well as from the plasma samples of vaccinated individuals on day 14 (*n* = 30) and day 28 (*n* = 30). The pairwise correlation coefficient (ρ) was determined using Spearman’s rank correlation.

As the levels of S RBD-specific IgM, IgA, and IgG antibodies significantly increased in patients after their recoveries from COVID-19, and as only the IgA and IgG antibodies were predominantly observed after vaccination, we analyzed the correlations of the different classes of antibodies against the Wuhan strains and three SARS-CoV-2 variants. We observed no significant correlations between the S RBD-specific IgM and IgA antibodies on day 14 and day 28 (Fig. S4) in either the patients or the vaccinated group. Likewise, we did not observe a significant correlation between the levels of IgM and IgG antibodies on day 14 and day 28 (Fig. S5) in either the patients or the vaccinated group. Interestingly, we found significant correlations between the levels of IgA and IgG antibodies against all of the SARS-CoV-2 strains in the COVID-19 patients on day 14 and day 28, with the ρ and *P* values on day 14 as follows: Wuhan strain (ρ = 0.70, *P* = 0.010), Alpha variant (ρ = 0.63, *P* = 0.024), Delta variant (ρ = 0.77, *P* = 0.003), and Omicron variant (ρ = 0.68, *P* = 0.011) (Fig. 3A–D). In the COVID-19 patients on day 28, the ρ and *P* values were as follows: Wuhan (ρ = 0.75, *P* < 0.001), Alpha (ρ = 0.60, *P* = 0.003), Delta (ρ = 0.73, *P* < 0.001), and Omicron (ρ = 0.55, *P* = 0.008) (Fig. 3I–L). However, no correlation between IgA and IgG was observed in the CoronaVac-vaccinated individuals at any time point post-enrollment (Fig. 3E–H and M–P).

10.1128/msphere.00465-22.4 FIG S4 Correlation between IgM and IgA antibody levels to S SARS-CoV-2 Wuhan and variants on day 14 (A–H) and day 28 (I–P) post-enrollment in COVID-19 patients and in CoronaVac-vaccinated individuals. (A, E, I, and M), IgM versus IgA antibody levels against the Wuhan strain. (B, F, J, and N), IgM versus IgA antibody levels against the Alpha variant. (C, G, K, and O), IgM versus IgA antibody levels against the Delta variant. (D, H, L, and P), IgM versus IgA antibody levels against the Omicron variant. Download FIG S4, DOCX file, 0.2 MB. Copyright © 2023 Poolchanuan et al. 2023 Poolchanuan et al. https://creativecommons.org/licenses/by/4.0/ This content is distributed under the terms of the Creative Commons Attribution 4.0 International license.

10.1128/msphere.00465-22.5 FIG S5 Correlation between IgM and IgG antibody levels to S SARS-CoV-2 Wuhan and variants on day 14 (A–H) and day 28 (I–P) post-enrollment in COVID-19 patients and in CoronaVac-vaccinated individuals. (A, E, I, and M), IgM versus IgG antibody levels against the Wuhan strain. (B, F, J, and N), IgM versus IgG antibody levels against the Alpha variant. (C, G, K, and O), IgM versus IgG antibody levels against the Delta variant. (D, H, L, and P), IgM versus IgG antibody levels against the Omicron variant. Download FIG S5, DOCX file, 0.2 MB. Copyright © 2023 Poolchanuan et al. 2023 Poolchanuan et al. https://creativecommons.org/licenses/by/4.0/ This content is distributed under the terms of the Creative Commons Attribution 4.0 International license.

### Correlation of the different IgG subclasses of antibody responses to the S RBD of SARS-CoV-2 variants in COVID-19 patients and in CoronaVac-vaccinated individuals.

**FIG 4:**
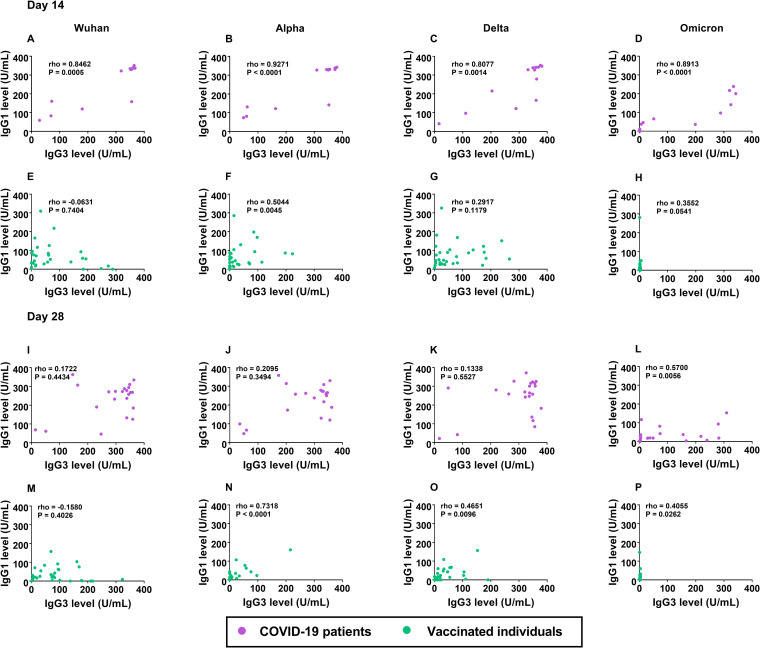
Correlation between IgG1 and IgG3 antibody levels to S SARS-CoV-2 Wuhan and variants on day 14 (A–H) and day 28 (I–P) in COVID-19 patients and in CoronaVac-vaccinated individuals. (A, E, I, and M) IgG1 versus IgG3 antibody against the Wuhan strain. (B, F, J, and N) IgG1 versus IgG3 antibody against the Alpha variant. (C, G, K, and O) IgG1 versus IgG3 antibody against the Delta variant. (D, H, L, and P) IgG1 versus IgG3 antibody against the Omicron variant. Antibodies were determined from the plasma samples of COVID-19 patients on day 14 (*n* = 13), day 28 (*n* = 22) as well as from the plasma samples of vaccinated individuals on day 14 (*n* = 30) and day 28 (*n* = 30). The pairwise correlation coefficient (ρ) was determined using Spearman’s rank correlation.

Our study revealed that IgG1 and IgG3 were predominantly elevated in COVID-19 patients and in vaccinated individuals on day 14 and that they declined on day 28. Therefore, we analyzed the correlation between the IgG1 and IgG3 antibodies to the SARS-CoV-2 variants on day 14 and day 28 in the specified groups (Fig. 4). Interestingly, on day 14 after enrollment, the IgG1 levels against all of the SARS-CoV-2 strains were highly correlated with the IgG3 levels, with the rho (ρ) and *P* values as follows: Wuhan strain (ρ = 0.85, *P* < 0.001), Alpha variant (ρ = 0.93, *P* < 0.001), Delta variant (ρ = 0.81, *P* = 0.001), and Omicron variant (ρ = 0.89, *P* < 0.001) (Fig. 4A–D). In contrast, no significant correlation between the IgG1 and IgG3 antibodies to the SARS-CoV-2 variants was observed on day 14 in the vaccinated group after the second dose of CoronaVac vaccination (Fig. 4E–H). A significant correlation was not found between the levels of the IgG1 and IgG3 antibodies against the SARS-CoV-2 variants on day 28 in the patients after the onset of symptoms (Fig. 4I–4L) or in the vaccinated group (Fig. 4M–4P). Furthermore, we observed no correlations between the levels of IgG1 and IgG4 or between the levels of IgG3 and IgG4 in the COVID-19 patients on day 14 or on day 28 (Fig. S6).

10.1128/msphere.00465-22.6 FIG S6 The correlation between IgG1 and IgG4 antibody levels to S SARS-CoV-2 Wuhan and variants on day 14 (A–D) and day 28 (E–H) as well as the correlation between IgG3 and IgG4 on day 14 (I–L) and day 28 (M–P) post-enrollment in COVID-19 patients. (A, E, I, and M), IgG1 versus IgG4 as well as and IgG3 versus IgG4 antibody levels against the Wuhan strain. (B, F, J, and N), IgG1 versus IgG4 as well as IgG3 versus IgG4 antibody levels against the Alpha variant. (C, G, K, and O), IgG1 versus IgG4 as well as IgG3 versus IgG4 antibody levels against the Delta variant. (D, H, L, and P), IgG1 versus IgG4 as well as IgG3 versus IgG4 antibody levels against the Omicron variant. Download FIG S6, DOCX file, 0.3 MB. Copyright © 2023 Poolchanuan et al. 2023 Poolchanuan et al. https://creativecommons.org/licenses/by/4.0/ This content is distributed under the terms of the Creative Commons Attribution 4.0 International license.

### Seropositivity of the different classes of antibodies in the COVID-19 patients.

**FIG 5:**
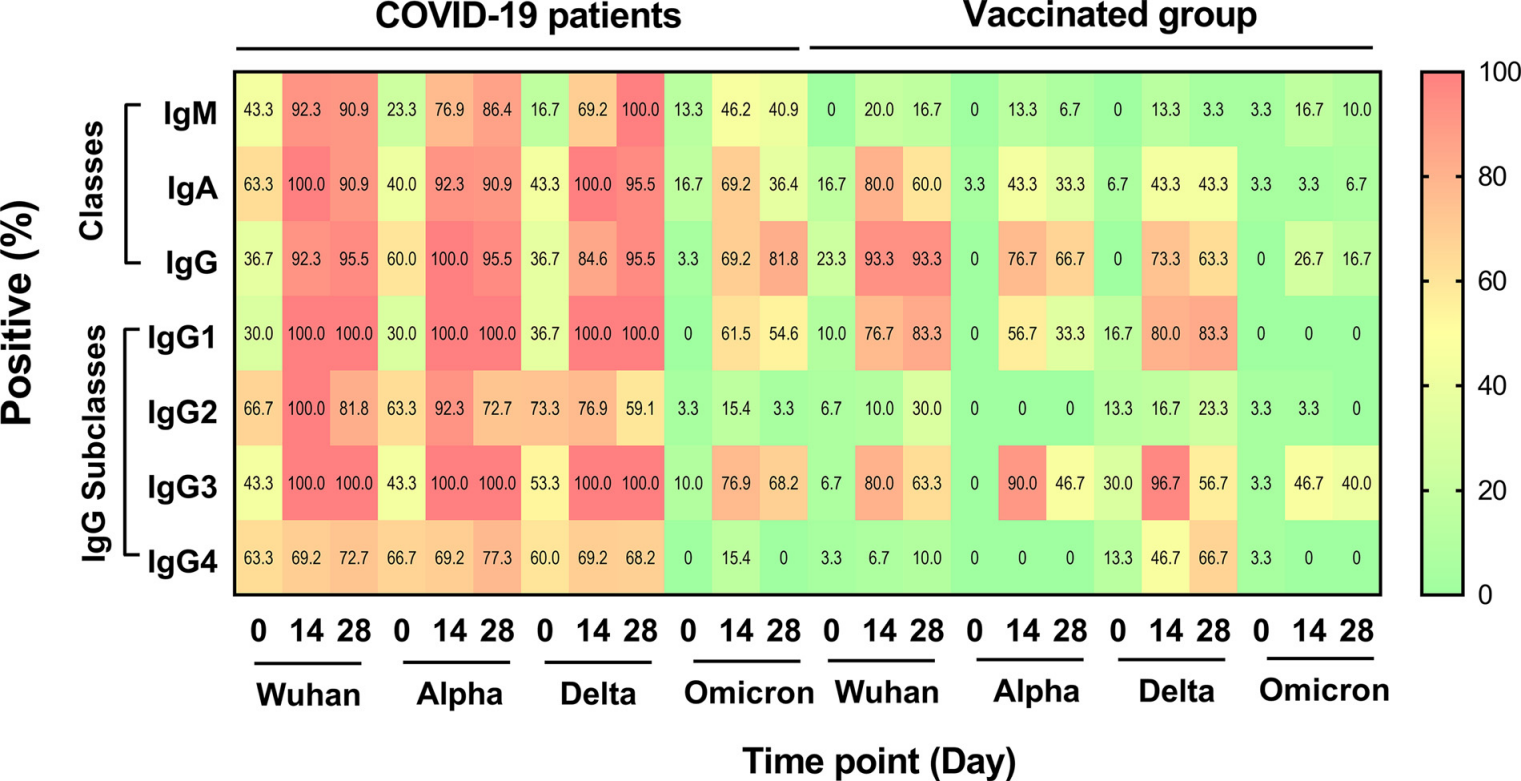
Seropositivity of different classes and IgG subclasses of anti-S RBD antibodies of SARS-CoV-2 variants in COVID-19 patients and in vaccinated individuals. The antibody levels were determined in plasma samples by ELISA. The patients were enrolled at the time of diagnosis (day 0), and blood was collected on day 0 (*n* = 30), day 14 (*n* = 13), and day 28 (*n* = 22). The vaccinated individuals were enrolled before vaccination (day 0) (*n* = 30). Blood samples were obtained on day 14 (*n* = 30) and day 28 (*n* = 30) after the second dose of vaccination. A high positive rate is shown in red, whereas a low positive rate is shown in green. Yellow, orange, and red for each block represent positive rates of antibody at 5.55, 8.91, 23.76, 6.57, 6.51, 11.08, and 8.57 U/mL for IgM, IgA, IgG, IgG1, IgG2, IgG3, and IgG4, respectively.

Using the ELISA cutoff values, the seropositivity of the different classes and IgG subclasses of antibodies against the Wuhan strain and the different VOCs were calculated and are presented as percentages in a heat map (Fig. 5).

Among the COVID-19 patients at day 0, the IgM, IgA, and IgG seropositivity was 36.7% to 63.3% for the Wuhan strain, 23.3% to 60.0% for the Alpha variant, 16.7% to 43.3% for the Delta variant, and 3.3% to 16.7% for the Omicron variant (Fig. 5). On day 14 after enrollment, the seropositivity of the IgM, IgA, and IgG antibodies against the S RBD of the different SARS-CoV-2 strains increased to 92.3% to 100% for the Wuhan strain, 76.9% to 100% for the Alpha variant, and 69.2% to 100% for the Delta variant. The seropositivity against the Omicron variant also increased to a lesser degree, with the range of IgM, IgA, and IgG being 46.2% to 69.2%. On day 28, the seropositivity of these antibodies against the Wuhan, Alpha, and Delta variants did not differ from those of day 14, ranging from 90.9% to 95.5% (Wuhan), 86.4% to 95.5% (Alpha), and 95.5% to 100% (Delta). However, we observed an increased seropositivity of the IgG anti-S RBD antibody against the Omicron variant on day 28, compared to day 14 (81.8% versus 69.2%), but decreased seropositivity for the IgM (40.9% versus 46.2%) and IgA (36.4% versus 69.2%) anti-S RBD antibodies.

### Seropositivity of the different classes of antibodies in the CoronaVac-vaccinated individuals.

The seropositivity rates of pre-vaccinated individuals (day 0) were used as a baseline for all of the classes of antibodies. On day 14, after receiving two doses of CoronaVac vaccination, the rate of IgM against the SARS-CoV-2 Wuhan strain and all of the variants was low, ranging from 13.3% to 20% (Fig. 5). In contrast, the IgA and IgG seropositivity of vaccinated individuals against the Wuhan, Alpha, and Delta variants increased on day 14. The positive rates for IgA against the Wuhan, Alpha, and Delta variants were 80%, 43.3%, and 43.3%, respectively, and those of IgG were 93.3%, 76.7%, and 73.3%, respectively. The positive rates of IgA and IgG against the Omicron variant on day 14 were low (3.3% and 26.7%, respectively). On day 28 after vaccination, the positive rates of the S RBD-specific IgM antibody against all of the SARS-CoV-2 variants were low, decreasing from those measured on day 14 (3.3% to 16.7%) (Fig. 5). The positive rates of IgA on day 28 decreased from those measured on day 14 for the Wuhan strain (60% versus 80%) and for the Alpha variant (33.3% versus 43.3%). The positive rate of the specific IgG antibody against the Wuhan strain persisted on day 28, compared to day 14 (93.3% versus 93.3%). However, we observed decreased levels of the IgG antibody against the Alpha (66.7% versus 76.7%) and Delta (63.3% versus 73.3%) variants.

### Seropositivity of the IgG subclasses of antibodies in COVID-19 patients.

As our results revealed high total IgG levels in both COVID-19 patients and vaccinated individuals, we further investigated the seropositivity rates of the plasma IgG subclasses against the Wuhan strain and the three VOCs in the COVID-19 patients and compared them with those of the CoronaVac-vaccinated individuals (Fig. 5).

On day 0, the COVID-19 patients had detectable seropositivity for the IgG1, IgG2, IgG3, and IgG4 S RBD-specific antibodies, ranging from 30% to 66.7% against the Wuhan strain, 30% to 66.7% against the Alpha variant, and 36.7% to 73.3% against the Delta variant, but the positive rates of these antibodies against the Omicron variant ranged from only 0% to 10% (Fig. 5). On day 14, we observed that all IgG subclass seropositivity rates against the Wuhan strain as well as the Alpha and Delta variants were high. The seropositivity rates were 100% for IgG1 and IgG3 on day 14, and these rates persisted until day 28 after infection. The seropositivity rates for IgG2 and IgG4 were lower than those of IgG1 and IgG3, and that of IgG2 decreased over time (day 14 and day 28: 100% versus 81.8% for the Wuhan strain, 92.3% versus 72.7% for the Alpha variant, and 76.9% versus 59.1% for the Delta variant). The seropositivity of IgG4 increased slightly or not changed on day 28 (day 14 and day 28: 69.2% versus 72.7% for the Wuhan strain, 69.2% versus 77.3% for the Alpha variant, and 69.2 versus 68.2% for the Delta variant). Compared to day 0, the seropositivity rates on day 14 and day 28 against the Omicron variant moderately increased for IgG1 (0 versus 61.5% versus 54.6%) and for IgG3 (10.0% versus 76.9% versus 68.2%). The seropositivity rates on day 14 and day 28 were low for IgG2 (day 0, day 14, and day 28: 3.3% versus 15.4% versus 3.3%) and IgG4 (day 0, day 14, and day 28: 0 versus 15.4% versus 0).

### Seropositivity rates of the IgG subclasses of antibodies in the CoronaVac-vaccinated individuals.

In the CoronaVac-vaccinated individuals, the seropositivity rates on day 14 were detected for IgG1 and IgG3 against the Wuhan strain as well as the Alpha and Delta variants (IgG1: 76.7%, 56.7%, and 80%, respectively; IgG3: 80%, 90%, and 96.7%, respectively), but the positive rates against the Omicron variant were 0% for IgG1 and 46.7% for IgG3 (Fig. 5). The positive rates for IgG1 against all of the VOCs on day 28 were either comparable to those on day 14 or decreased in comparison to those on day 14. Only 40.0% of the CoronaVac-vaccinated group was seropositive for IgG3 on day 28 against the Omicron variant. The IgG1, IgG2, and IgG4 values of the CoronaVac-vaccinated individuals were negative against the Omicron variant.

### Antibody levels against the SARS-CoV-2 variants in the COVID-19 patients with and without pneumonia.

24 COVID-19 patients (80%) had pneumonia (Table 1). Therefore, compared the immune responses between the two groups on day 0. The levels of IgM, IgA, and IgG antibodies against the Wuhan strain as well as the Alpha and Delta variants were not different between the patients with and without pneumonia. We found that only the IgM level against Omicron was significantly higher in the patients with pneumonia, compared to the patients without pneumonia (median [IQR], 2.4 [0.3 to 7.5] versus 0 [0 to 2], *P* = 0.003) (Fig. S7). Due to a loss of follow-up, the number of patients without pneumonia on day 14 was low (*n* =1). Therefore, we did not compare antibody levels between the patients with and without pneumonia on days 14 and 28.

10.1128/msphere.00465-22.7 FIG S7 Levels of day 0 IgM, IgA, and IgG antibodies against the S RBD proteins of SARS-CoV-2 variants in COVID-19 patients with non-pneumonia (*n* = 6) and pneumonia (*n* = 24). Antibodies were determined from the plasma of patients by ELISA. (A) Antibodies against the Wuhan strain. (B) Antibodies against the Alpha variant. (C) Antibodies against the Delta variant. (D) Antibodies against the Omicron variant. Download FIG S7, DOCX file, 0.1 MB. Copyright © 2023 Poolchanuan et al. 2023 Poolchanuan et al. https://creativecommons.org/licenses/by/4.0/ This content is distributed under the terms of the Creative Commons Attribution 4.0 International license.

### Neutralizing antibodies against the SARS-CoV-2 variants in COVID-19 patients and in vaccinated individuals.

**FIG 6:**
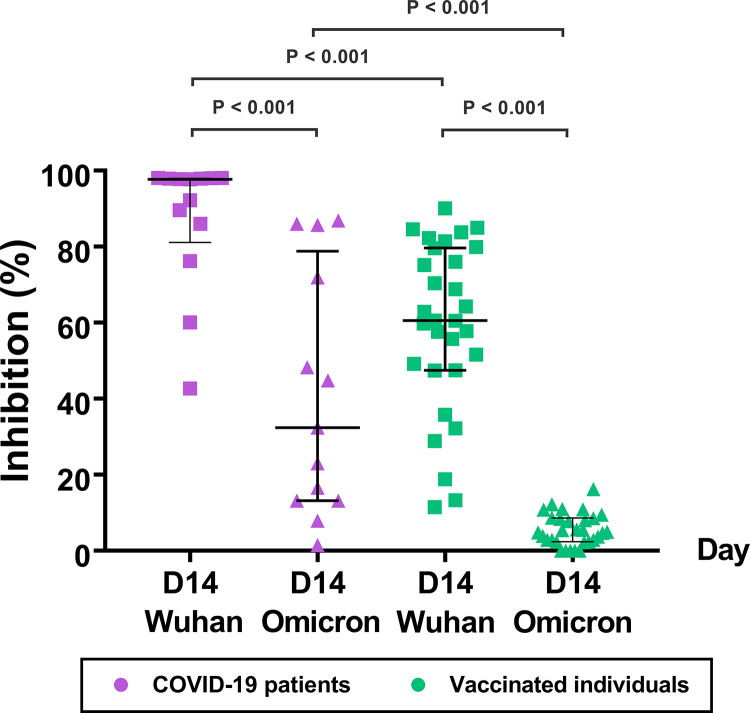
Neutralizing antibody (% inhibition) against the S SARS-CoV-2 Wuhan strain and Omicron variant in COVID-19 patients (*n* = 13) and in CoronaVac-vaccinated individuals (*n* = 30). The antibody levels were determined in plasma samples collected on day 14 using a cPass kit. A percent inhibition of <30 was interpreted as negative.

To determine the total neutralizing activity of antibodies (NAbs) in the plasma samples of the COVID-19 patients and the CoronaVac-vaccinated individuals, we used a cPass SARS-CoV-2 Neutralization Antibody Detection Kit, which is also known as a SARS-CoV-2 Surrogate Virus Neutralization Test (sVNT) Kit. The sVNT measured the NAbs levels in the plasma that blocked the interaction between the RBDs and the human ACE2 receptor (Fig. 6). We used two different SARS-CoV-2 RBDs (Wuhan and Omicron) conjugated with horseradish peroxidase (HRP) in the assays. We found that the plasma of the COVID-19 patients had high NAbs that blocked the Wuhan RBD at a median inhibition of 97.7% (IQR: 81.1% to 98.0%) and the Omicron RBD at a median inhibition of 32.4% (IQR: 13.2% to 78.8%) (*P* < 0.001).

In contrast, the plasma of the vaccinated individuals showed individual variation and lower blocking activity against the Wuhan RBD with a median inhibition of 60.6% (IQR: 47.5 to 79.7) (patients versus vaccinated individuals, *P* < 0.001). The vaccinated group did not show blocking activity against the Omicron RBD (median inhibition, 4.9% [IQR: 2.4 to 8.6]) (Wuhan versus Omicron, *P* < 0.001). The data suggest that the patients had higher levels of NAbs against the Wuhan strain and the Omicron variant than did the vaccinated individuals.

### Correlation of classes, subclasses, and neutralizing antibodies in COVID-19 patients and in CoronaVac-vaccinated individuals.

**FIG 7:**
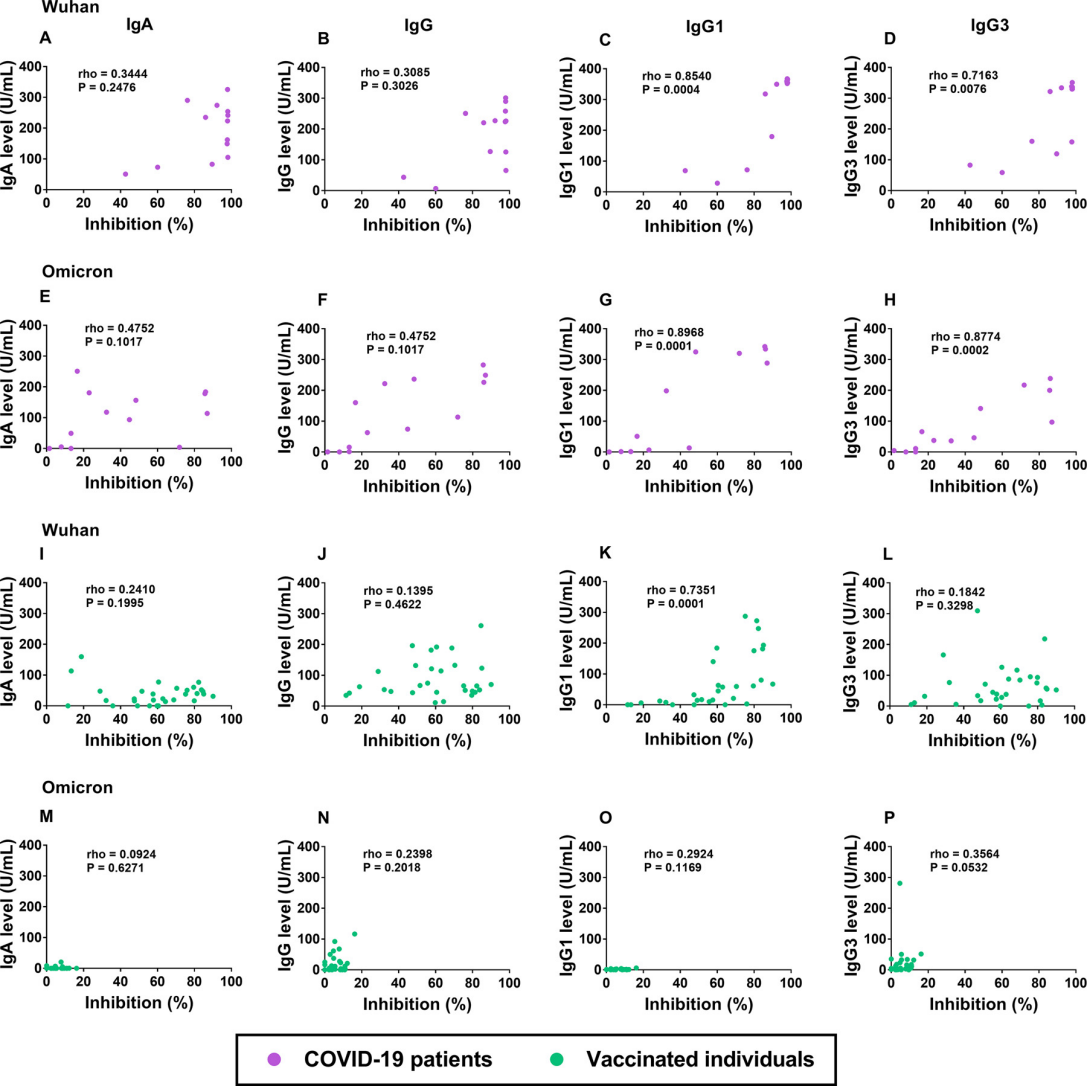
Correlation between neutralizing antibodies (% inhibition) and S RBD specific antibodies on day 14 against the Wuhan strain (A–D, I–L) and Omicron variant (E–H, M–P) in COVID-19 patients (*n* = 13) (A–H) and in CoronaVac-vaccinated individuals (*n* = 30) (I–P). (A, E, I, and M) Neutralizing antibody versus IgA antibody. (B, F, J, and N) Neutralizing antibody versus IgG antibody. (C, G, K, and O) Neutralizing antibody versus IgG1 antibody. (D, H, L and P) Neutralizing antibody versus IgG3 antibody. The pairwise correlation coefficient (ρ) was determined using Spearman’s rank correlation.

As the levels of the S RBD-specific IgA, IgG, IgG1, and IgG3 antibodies were detectable in both patients after recovery and after vaccination, we analyzed the correlations between the NAbs on day 14 and the levels of these antibodies against the Wuhan strain and the Omicron variant (Fig. 7). Interestingly, in the COVID-19 patients, we observed high correlations between the NAbs and the S RBD-specific IgG1 antibodies against the Wuhan strain (ρ = 0.85, *P* < 0.001) and the Omicron variant (ρ = 0.90, *P* < 0.001) (Fig. 7C and G) as well as the S RBD-specific IgG3 antibodies against the Wuhan strain (ρ = 0.72, *P* = 0.008) and the Omicron variant (ρ = 0.88, *P* < 0.001) (Fig. 7D and H). The vaccinated individuals showed a significant correlation only between the NAbs and the S RBD-specific IgG1 (but not IgG3) against the Wuhan RBD (Fig. 7K and L). We did not observe significant correlations between the NAbs and IgA or between the NAbs and the total IgG antibodies against the two SARS-CoV-2 strains in the COVID-19 patients (Fig. 7A, B, E, and F) or in the vaccinated individuals (Fig. 7I, J, M, and N).

## DISCUSSION

The main findings of this study are as follows: (i) the plasma samples of the COVID-19 patients showed higher and broader classes and subclasses of S RBD-specific antibody responses with higher neutralizing activities to the closely related SARS-CoV-2 lineages (Wuhan, Alpha, and Delta) than did those of healthy individuals who were vaccinated with CoronaVac; (ii) the median antibody levels peaked in 2 weeks and started to decline in a month; (iii) IgG1 and IgG3 were the major IgG subclasses, and they increased in both patients and CoronaVac-vaccinated individuals; (iv) IgA levels were correlated with IgG levels, and IgG1 levels were correlated with IgG3 levels in COVID-19 patients, but no such correlations were observed in CoronaVac-vaccinated individuals; (v) the levels of all antibodies against the S RBD of the Omicron variant were low in both COVID-19 patients and vaccinated individuals; and (vi) the percent inhibition of the NAbs in the plasma of COVID-19 patients correlated with the IgG1 and IgG3 levels, whereas the percent inhibition in the plasma of CoronaVac-vaccinated individuals correlated with the IgG1 level only.

High seropositivity rates were readily detected in the COVID-19 patients when they had clinical symptoms, and the seropositivity rates increased to their highest levels in 2 weeks. In this study, the ranges of seropositivity against the S RBD of the Wuhan strain as well as the Alpha and Delta variants at 2 weeks after the onset of symptoms were 69.2% to 92.3% for IgM, 92.3% to 100% for IgA, and 84.6% to 100% for IgG. Our results are consistent with those reported in China and Spain (7, 16, 23). The correlation between IgA and IgG on day 14 and day 28 after the onset of symptoms in our study agreed with the results presented in previous reports (15, 18, 24, 25). We observed decreased levels of these antibodies on day 28 but did not follow up with the patients after day 28. However, other reports have demonstrated that the IgM level decreased rapidly to 0% at week 13, but the positivity rates of IgA and IgG remained high at 4 to 6 months after the onset of disease (8, 13, 16, 18, 19).

The high levels of the IgA and IgG antibodies indicate sustained humoral immunity development in convalescent patients (16, 20). A number of patients in our study had existing IgG antibodies from previous vaccinations, but not at a sufficient level to prevent infection. Many studies have shown that the level of IgG and the dynamics of antibodies are associated with the durability of infection and disease severity (19, 23). The results of our neutralization-antibody experiments indicate that the NAbs occurring in patients following previous infections can block closely related infecting strains, but they may not effectively block the entry of the new SARS-CoV-2 Omicron variant.

CoronaVac is an inactivated viral vaccine produced from the Wuhan strain, and it was the first vaccine immunized for people in Thailand in early 2020, as in many Asian countries and Africa. Our study demonstrated that the ranges of seropositivity against the Wuhan strain as well as the Alpha and Delta variants after 14 days of vaccination were 13.3% to 20% for IgM, 43.3% to 80% for IgA, and 73.3% to 93.3% for IgG. Consistent with our results, previous studies in China and Brazil showed that same rates on day 14 after the second dose of CoronaVac (7–9, 26). With two doses of this vaccine, no IgA and IgG correlation was observed in our vaccinated group, suggesting that a complex of the class switching of antibodies is induced by CoronaVac vaccination. In our study, the antibody levels of the vaccinated individuals declined on day 28. Xu et al. observed that the IgM, IgA, and IgG antibodies decayed on day 160 after the CoronaVac vaccination (8). Our study indicated that the IgM antibody to the S RBD was less detectable in the vaccinated individuals who received the CoronaVac vaccine than in the COVID-19 patients. IgM is considered to be a part of innate immunity and natural antibodies (27). Thus, the IgM level in vaccinated individuals slightly increased and rapidly decreased to the baseline during the two doses of vaccination. Interestingly, our data demonstrate significantly higher levels of IgG recognizing the S RBD of the Wuhan strain as well as the Alpha and Delta variants, compared to the level of the IgM antibody. Although the median level of the IgA antibody was detectable, it was significantly lower than that of IgG. IgA is a predominant immunity at the mucosal surfaces where the pathogen-host interaction occurs during infection, which may not appear during vaccination by injections (28). Other studies have also shown that IgA persists longer than does the IgM antibody and that circulating IgA may become detectable during the advanced stages of infection and vaccination (18, 22, 25). Overall, these results suggest that CoronaVac vaccination may not systemically induce effective IgA and IgM antibodies against SARS-CoV-2.

Our heat map analysis showed the longitudinal data of the diverse subclasses of IgG antibody responses to the S RBD in the COVID-19 patients and in the vaccinated individuals. In the COVID-19 patients, the seropositivity rates at 14 days after the onset of symptoms were 100% for IgG1, 76.9% to 100% for IgG2, 100% for IgG3, and 69.2% for IgG4. Consistent with our study, studies in Brazil, Israel, and the USA reported the same seropositivity rates after 14 days of disease diagnosis (8, 29, 30). Our correlation analysis showed that the IgG1 level was correlated with the IgG3 level, supporting the same results of previous studies in the USA and our previous study in Thailand (24, 29). These studies also detected high IgG1 and IgG3 levels in severe patients, compared to patients with mild and moderate symptoms (29, 31). The positivity rates of the IgG subclasses in our COVID-19 patients on day 28 were 100% for IgG1, 59.1% to 81.8% for IgG2, 100% for IgG3, and 68.2% to 77.3% for IgG4. The same positive rates were found in a previous report in Brazil (32). The COVID-19 patients were found to have an enrichment of IgG1 with afucosylated Fc glycans, which could heighten the affinity of IgG to the Fcγ receptor, an effector function on monocytes, macrophages, and NK cells. These cells are responsible for proinflammatory cytokine production and cytotoxic effector cell activity (29–31, 33, 34). High levels of IgG1, IgG3, and IgG4 were detected in patients with severe disease and comorbidities, which were positively correlated with the production of interleukin-6 (IL-6) and the tumor necrosis factor (TNF) (29, 31–33, 35). Our results provided additional evidence that the RBD-specific IgG1 and IgG3, but not the total IgG antibodies, in the plasma of the convalescent patients correlated with neutralization activity. The total IgG contained IgG subclasses other than IgG1 and IgG3 that might have poor binding affinity to block RBD from binding to the host ACE protein. Our results are consistent with those of previous studies that have reported that IgG1 and IgG3 were the predominant IgG subclasses detected after COVID-19 and vaccination. IgG1 and IgG3 are the strong mediators involved in the response to the protein antigen (29–32). It is likely that SARS-CoV-2 infections activate humoral immune responses in T-cell-dependent and T-cell independent manners; thus, multiple classes and subclasses of antibodies were detected in the patients. In our study, day 0 was the first day on which patients were diagnosed and confirmed via polymerase chain reaction (PCR). Some patients might have been infected for several days and have already developed a range of humoral immune responses to the S RBD antigens. Half of our patients had a long duration of hospital stay (>10 days) and involvement of several organs, including infections in the lung (80%), liver (33.3%), and kidney (6.7%), and more than half of the patients had comorbidities. Many of these host factors may have led to the induction of different IgG subclasses of antibodies in response to SARS-CoV-2 infection. However, the associations between these host variables and the subclasses of antibodies require further studies.

This study demonstrated that the IgG response to CoronaVac vaccination was primarily restricted to the IgG1 and IgG3 subclasses. After 14 days after the second dose of the CoronaVac vaccination, the seropositivity ranges were 56.7% to 80% for IgG1, 0% to 16.7% for IgG2, 76.9% to 96.7% for IgG3, and 0% to 46.7% for IgG4. We observed no correlation between IgG1 and IgG3 on day 14 after the second dose of the CoronaVac vaccination. Only the IgG1 antibody of the CoronaVac-vaccinated individuals correlated with the neutralization function. The lack of correlation in the levels and neutralization functions between the two subclasses suggests that the increased IgG1 and IgG3 levels after vaccination probably represent fractions of switching IgG from IgM and might not correlatedly protect SARS-CoV-2 infection. We observed that different IgG subclasses similarly declined on day 28 after the CoronaVac vaccination. In contrast, a study on the mRNA vaccine showed that IgG1, IgG2, IgG3, and IgG4 increased approximately 100-fold, 8-fold, 50-fold, and 2-fold, respectively, after 14 days in people receiving the second dose of the Pfizer-BioNTech vaccine, and these IgG subclasses remained detectable on week 5 after vaccination (30). The results from this study, combined with those from other studies, indicate that different types of vaccination induce different pathways of humoral immunity against SARS-CoV-2 (22). Future studies are required to explore the causality of the different effector functions of these specific antibodies.

Importantly, our heat map analysis indicated that the seropositivity rates of the antibodies against the Omicron variant on day 14 in the COVID-19 patients were significantly lower than the rates of the antibodies against the other SARS-CoV-2 strains. Similarly, previous studies reported that the positive detection of neutralizing antibodies against the Omicron variant was low in COVID-19 patients (36). Evidence in patients with breakthrough infections who had been fully vaccinated with CoronaVac showed a 6.3-fold lower level of neutralizing antibody against the Omicron variant, compared to that against the Delta variant (37). Moreover, a study in Hong Kong also reported no detectable antibody to the Omicron variant in vaccinated individuals who received two doses of the CoronaVac vaccine (38). Our results in Thailand agree with these CoronaVac vaccination studies and suggest that COVID-19 and vaccination with original strains and related VOCs did not fully elicit a cross-reacting antibody against the S RBD of the Omicron variant. A high number of amino acid mutations in the S of the Omicron variant and subvariants can enhance the interaction between the RBDs and the electronegative human angiotensin-converting enzyme 2 (hACE2), which can lead to a higher transmission rate, immune evasion, and a reduction of vaccine effectiveness (6, 36, 39–41).

We noted interindividual variations in the levels and neutralization functions of the antibodies and IgG subclasses in different patients and vaccinated individuals. The results suggest that different pathways of humoral immune responses may be used in recognizing the S RBD antigens of VOCs. We found that the IgM level against the Omicron variant was significantly higher in patients with pneumonia compared to patients without pneumonia. Other studies have found an association of these antibody levels with age, the immune status of patients, the durability of infection, and the severity of disease (23, 29, 31). The different characteristics of individuals may explain the types and the levels of the antibody responses.

The limitations in this study are as follows: (i) the infected strains of SARS-CoV-2 were not determined in patients; (ii) quarantine after hospital discharge led to a lack of follow-up of some patients; (iii) the numbers of participants in our cohorts were relatively small; and (iv) the unit of antibody level was interpreted in-house and may not be comparable with the results of other publications that used different interpretative criteria. It is possible that the sequence of the Omicron RBD, which is 50 amino acids shorter than other RBDs, had some effect on the binding of antibodies in the ELISA and the neutralization assays. However, this study revealed distinct classes and subclasses of antibody responses against SARS-CoV-2 variants between COVID-19 patients and healthy individuals, post-CoronaVac vaccination. The distinct classes and subclasses of antibodies may play an important role in humoral immune protection and may inform the development of vaccines for COVID-19. As new variants are continuously emerging, the functions of the classes and subclasses of antibodies against new variants require further validation in larger studies.

## MATERIALS AND METHODS

### Ethics statement.

This study was reviewed and approved by the Ethics Committees of the Faculty of Tropical Medicine, Mahidol University (approval number: MUTM 2021-019-01 and MUTM 2021-028-01). The study was performed under the principles of the Declaration of Helsinki (2008) and the International Conference on Harmonization (ICH) Good Clinical Practice (GCP) guidelines. Written informed consent was obtained from all study participants.

### Enrollment of COVID-19 patients.

We enrolled and collected plasma samples from 30 COVID-19 patients who were admitted to the Hospital for Tropical Diseases, Faculty of Tropical Medicine, Mahidol University, Bangkok, Thailand, between July and August of 2021. Blood samples were obtained on day 0 (*n* = 30), day 14 (*n* = 13), and day 28 (*n* = 22). Day 0 was the first day that COVID-19 was diagnosed and confirmed by a real-time reverse transcription-PCR (RT-PCR). The inclusion criteria were adult Thai patients aged ≥18 years with a positive result of RT-PCR for SARS-CoV-2 who could provide informed consent. The exclusion criteria were pregnancy or delivery in the past 9 months and the use of immune-modifying agents, anti-inflammatory agents, or cell depletion biological agents in the past 4 weeks. Due to a loss of follow-up at some points, the numbers of COVID-19 patients on day 14 and on day 28 were less than 30.

### Enrollment of CoronaVac-vaccinated individuals.

30 CoronaVac-vaccinated individuals were enrolled at the Hospital for Tropical Diseases, Faculty of Tropical Medicine, Mahidol University, Bangkok, Thailand, between May and June of 2021. Plasma samples were collected on day 0 (before the first dose of vaccination), day 14, and day 28 after the second dose of the CoronaVac vaccination. The duration between the first and second doses of vaccination was 21 to 28 days. The inclusion criteria of vaccinated individuals were healthy Thai adults aged ≥ 18 years who could provide informed consent. The exclusion criteria were pregnancy or delivery in the past 9 months, the use of immune-modifying agents, anti-inflammatory agents, or cell depletion biological agents in the past 4 weeks, having a chronic medical condition, having vigorous exercise in the past 24 h, the use of alcohol in the past 24 h, the administration of any immunoglobulins, the receipt of blood products in the past 4 months, having vaccinations in the past month; the administration of any research medicines or research vaccines in the past month, having any surgery plan within 6 months after the last immunization, and a history of COVID-19 infection. The samples taken before the first dose of vaccination on day 0 were used as the baseline controls in all of the immunoassays.

### Enzyme-linked immunosorbent assay (ELISA) to determine the specific antibodies to the SARS-CoV-2 variants.

The IgM, IgA, and IgG antibodies, as well as the IgG subclasses of antibodies (IgG1, IgG2, IgG3, and IgG4), that are specific to the recombinant S RBD of the SARS-CoV-2 Wuhan strain (Arg319-Ser591), as well as the Alpha (Arg319-Ser591 [N501Y]), Delta (Arg319-Ser591 [L452R, T478K]), and Omicron (BA.1) variants (Arg319-Phe541) (GenScript, USA), were determined by ELISA, as previously described, with some modifications (24). Plasma samples were used at a dilution of 1:100. The detection of secondary antibodies and horseradish peroxidase (HRP)-conjugated anti-human IgG subclasses (SouthernBiotech, USA) was determined at a dilution of 1:500, and anti-human IgM (Dako, USA), IgA (Invitrogen, USA), and IgG (Dako, USA) were used at dilutions of 1:1000, 1:2000, and 1:4000, respectively. Pooled plasma samples of 10 RT-PCR-confirmed COVID-19 patients were used as a positive control, and pooled plasma samples of 10 healthy donors were used as a negative control. Positive and negative controls, as well as assay diluent, were included in every assay plate. The values of these controls were within the mean ± 2 standard deviations (SD). All samples were performed once and analyzed in duplicate. In this study, an optical density (OD) value of 0.01 was interpreted as 1 unit (U)/mL of antibody level. The OD cutoff values for the IgM, IgA, and IgG antibodies were 5.55, 8.91, and 23.76 U/mL, and those for the IgG1, IgG2, IgG3, and IgG4 subclasses were 6.57, 6.51, 11.08, and 8.57 U/mL, respectively. The seropositivity was calculated from the number of patients who had antibody levels above the cutoff values, divided by the total number of patients, multiplies by one hundred.

### Electrochemiluminescence immunoassay (ECLIA) to determine the anti-nucleocapsid antibody to SARS-CoV-2.

The IgG antibody specific to the recombinant nucleocapsid (N) of SARS-CoV-2 (Roche, Switzerland) was determined via ECLIA, as described by the manufacturer. We used an Elecsys Anti-SARS-CoV-2 Kit that can detect infections of all of the SARS-CoV-2 variants. 30 pre-vaccination (day 0) plasma samples were incubated with biotinylated SARS-CoV-2-specific recombinant antigen and the same antigen labeled with a ruthenium complex to form a sandwich complex. Streptavidin-coated microparticles were added to the complex to generate a bond between the complex and the solid phase via a biotin and streptavidin interaction. The reaction mixture was aspirated into the measuring cell. A voltage was applied to the electrode, creating a chemiluminescent emission, which was analyzed using a Cobas E411 analyzer. The results were interpreted by comparing the electrochemiluminescence signal that was obtained from the samples with the signal of the cutoff value that was obtained from the calibration. The nonreactive results were a value range of the cutoff index (COI) that was <1.0. The internal control was provided by the manufacturer, and the pooled plasma samples of 10 vaccinated individuals were used as positive controls. All samples were performed once and analyzed in duplicate.

### SARS-CoV-2 neutralizing antibody detection.

The neutralizing antibody against the S RBD proteins of the SARS-CoV-2 variants was determined using a neutralization antibody detection kit, namely, cPass (GenScript, USA). The plasma samples and controls were diluted at 1:10 with sample dilution buffer. Horseradish peroxidase (HRP)-conjugated Wuhan (Arg319-Ser591) or Omicron (BA.1) (Arg319-Phe541) RBDs were diluted with HRB dilution buffer at a dilution of 1:1000. The diluted plasma samples and controls were mixed with the HRP-RBD solution at a volume ratio of 1:1 and were incubated at 37°C for 30 min. Then, 100 μL of the mixture were added to corresponding wells precoated with the human ACE2 receptor, and they were incubated at 37°C for 15 min. Then, the plate was washed 4 times, and 100 μL of 3,3′,5,5′-tetramethylbenzidine (TMB) were added and incubated for 15 min. The reaction was stopped via the addition of 50 μL of 1 N HCl. The OD was measured at 450 nm. The inhibition percentage was calculated by the subtraction of 1 and the OD value of the sample, divided by the OD value of the negative control. The subtracted value was multiplied by 100. The cutoff value for neutralization was 30%, as recommended by the manufacturer. All samples were performed once and analyzed in duplicate.

### Statistical analysis.

All of the statistical data were analyzed using GraphPad Prism version 7.0 (GraphPad Software Inc., San Diego, CA, USA). The Mann-Whitney test was used to compare the median differences in the data between groups. The pairwise correlation coefficient (ρ) was determined using Spearman’s rank correlation. A *P* value of <0.05 was considered to be indicative of a statistically significant result. The cutoff value for seropositivity was calculated from the mean + 2 standard deviations (SD) of 30 pre-vaccinated, healthy individuals.

### Data availability.

All of the data of this study are available in the article and the supplemental material.
