# Supplementary figures and images for: Dynamics of Different Classes and Subclasses of Antibody Responses to Severe Acute Respiratory Syndrome Coronavirus 2 Variants after Coronavirus Disease 2019 and CoronaVac Vaccination in Thailand
Source: mSphere. 2023 Jan 23;8(1):e00465-22. doi: 10.1128/msphere.00465-22 (PMC9942573; doi:10.1128/msphere.00465-22)

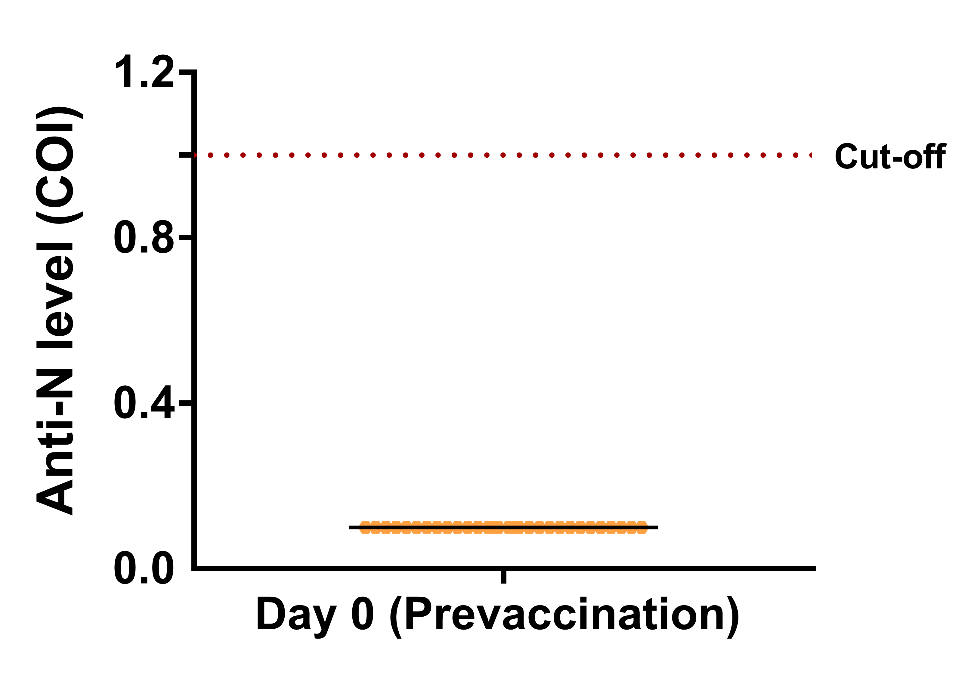


**Supplementary Figure S1**

Supplement: FIG S1 [file msphere.00465-22-s0001.docx]

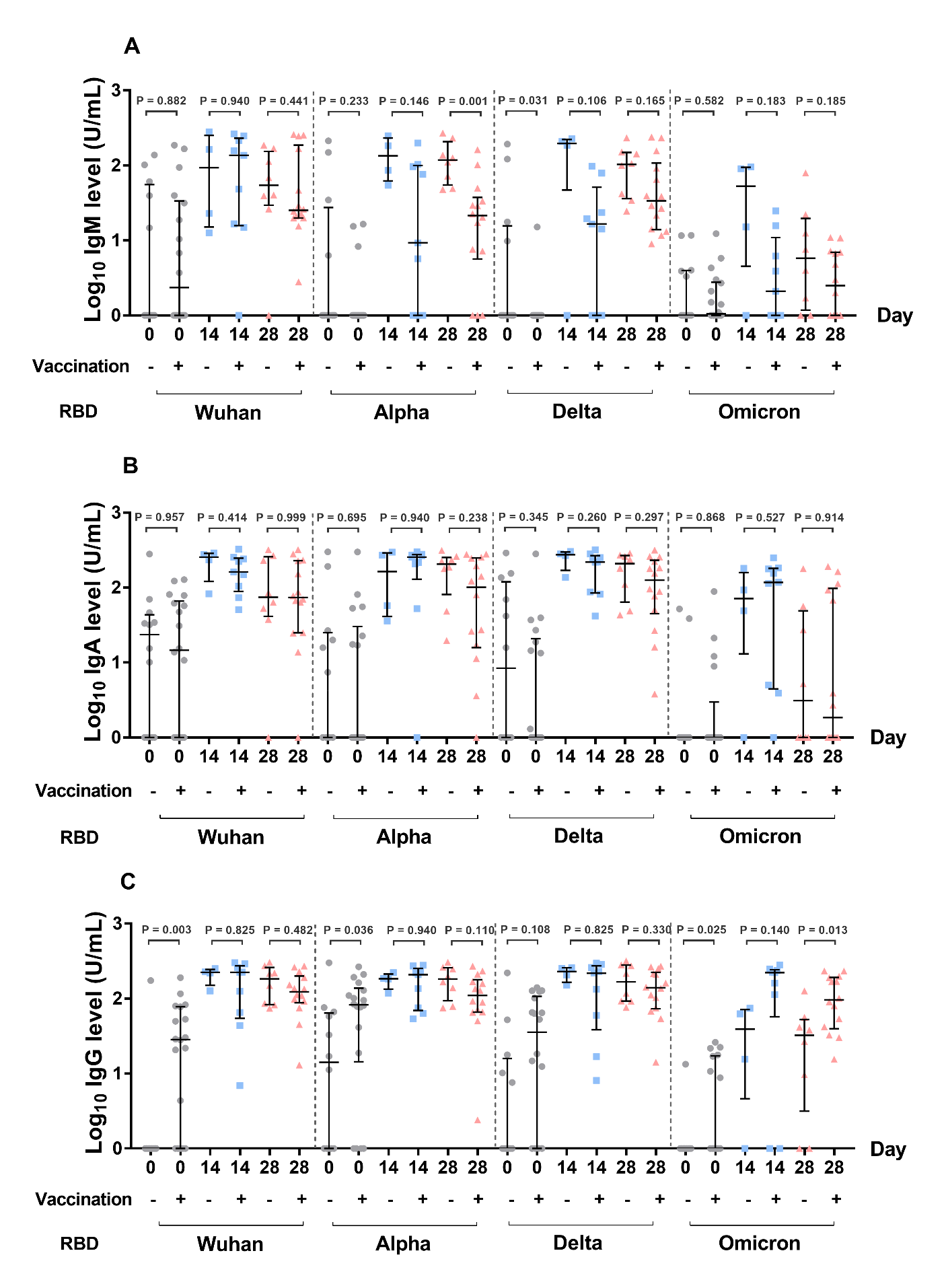
**Supplementary Figure S2**

Supplement: FIG S2 [file msphere.00465-22-s0002.docx]

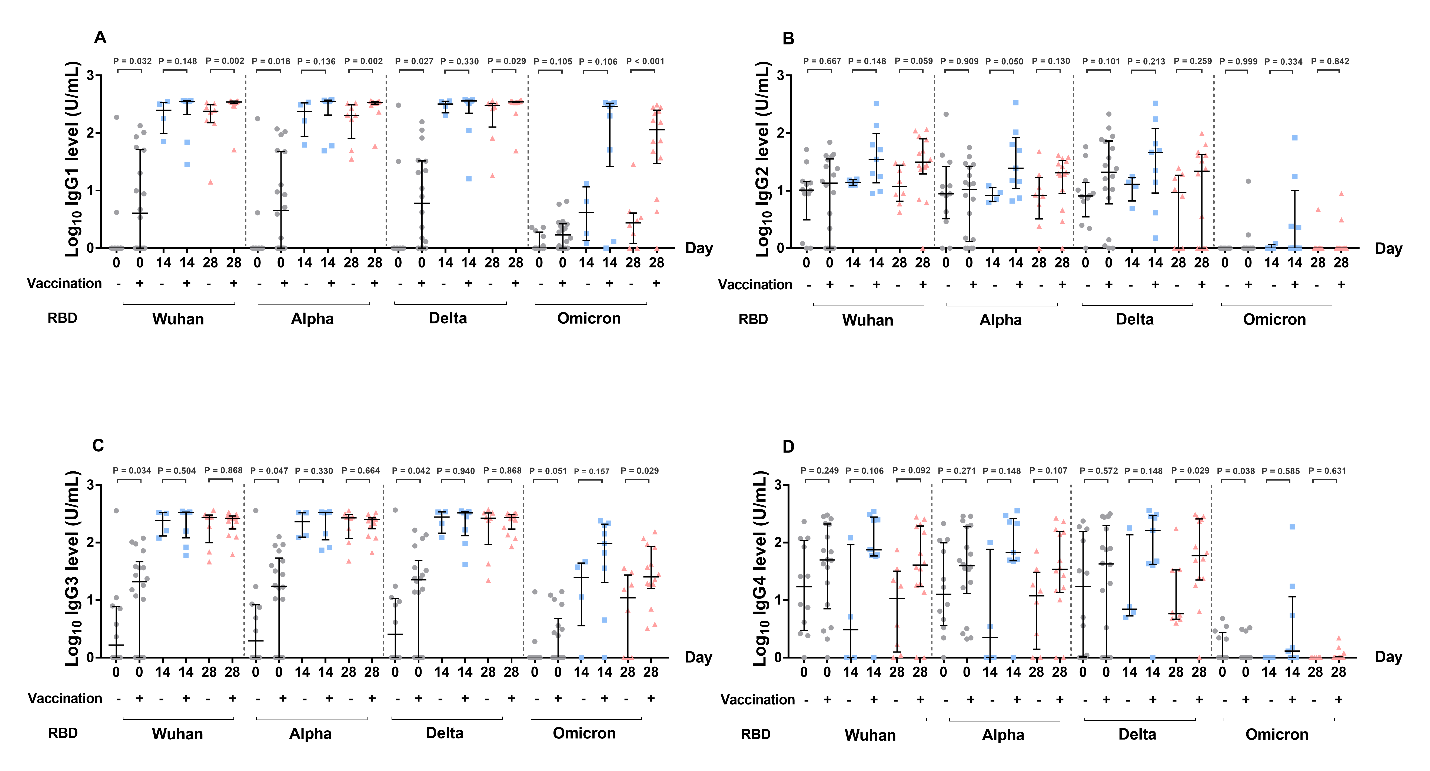


**Supplementary Figure S3**

Supplement: FIG S3 [file msphere.00465-22-s0003.docx]

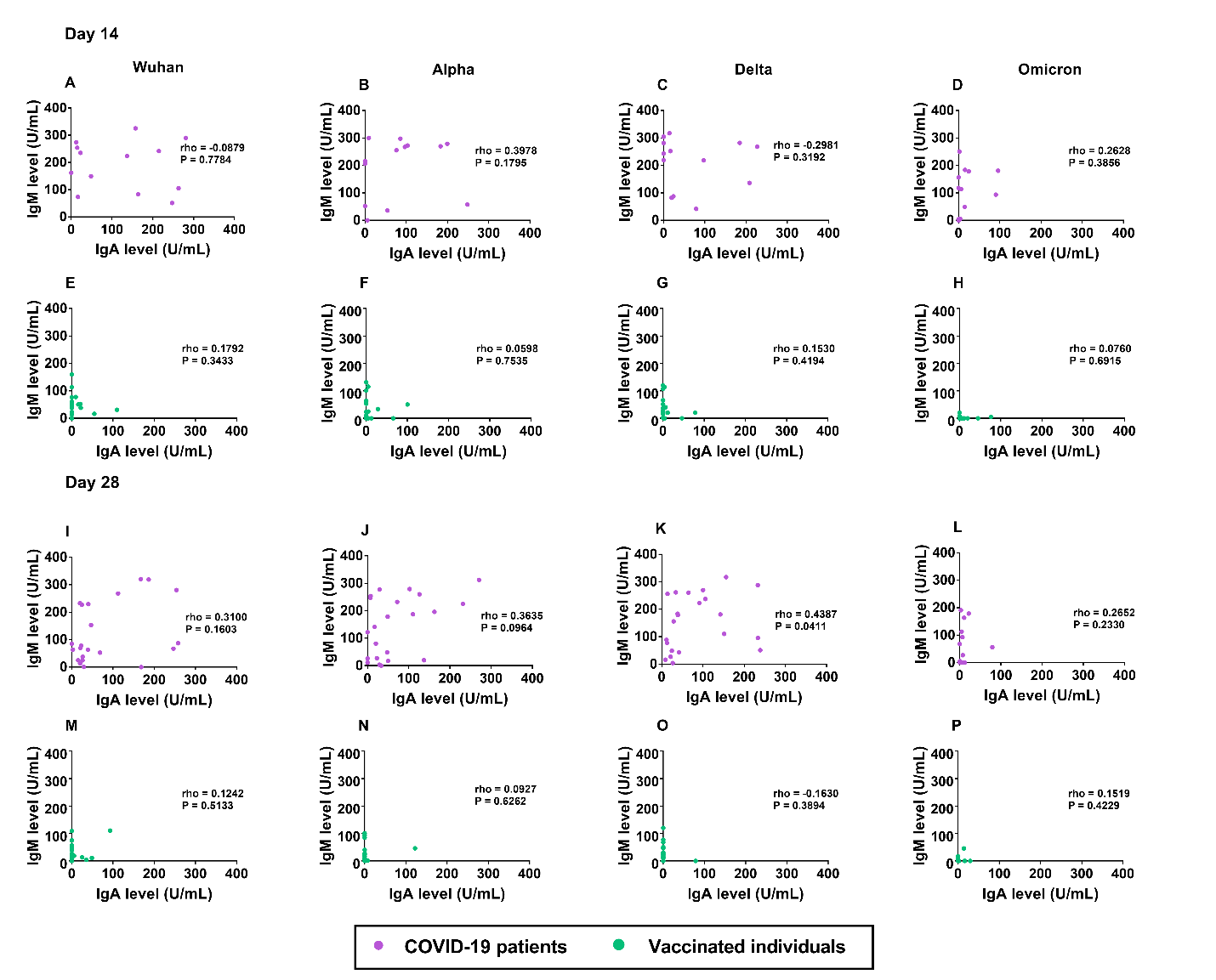


**Supplementary Figure S4**

Supplement: FIG S4 [file msphere.00465-22-s0004.docx]

**
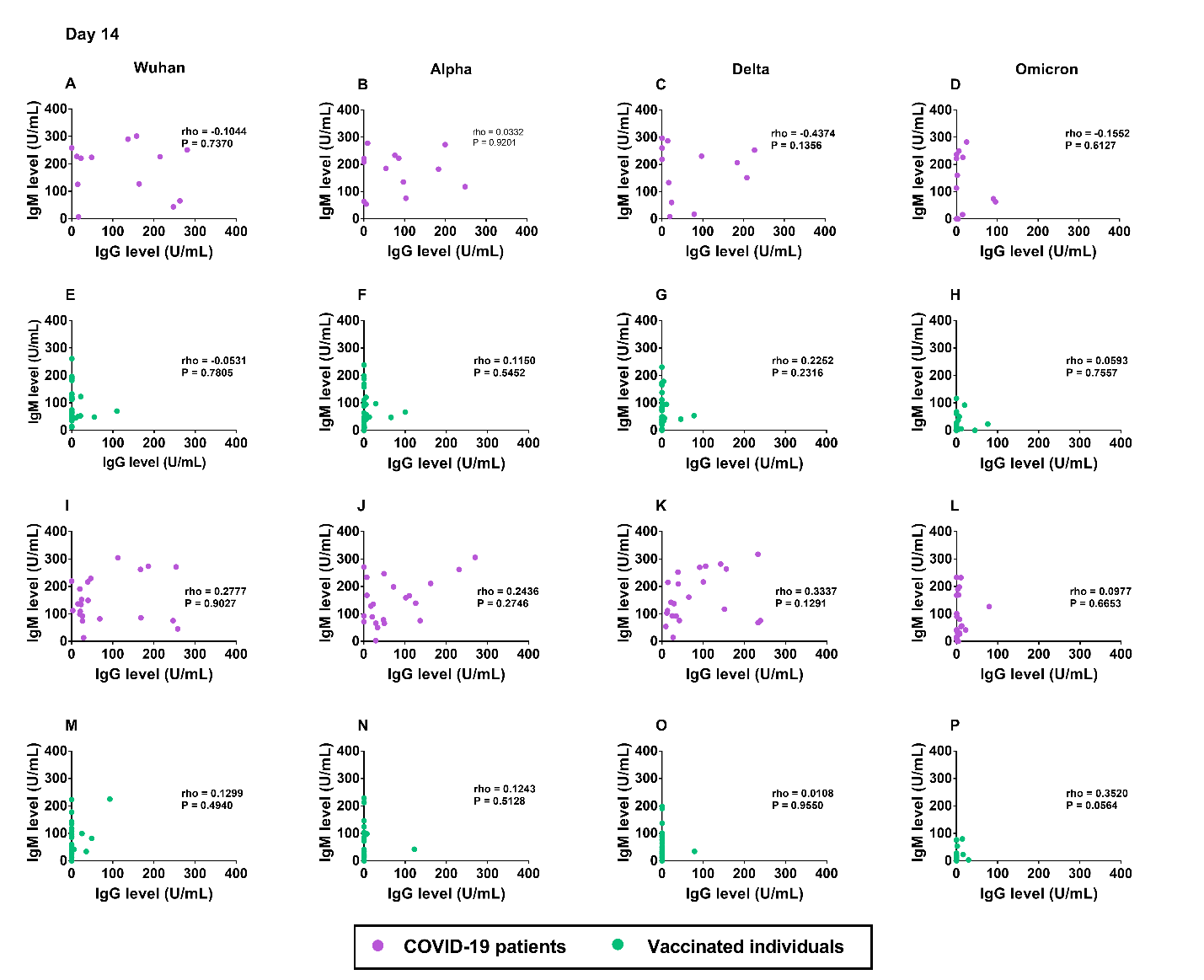
**

**Supplementary Figure S5**

Supplement: FIG S5 [file msphere.00465-22-s0005.docx]

**
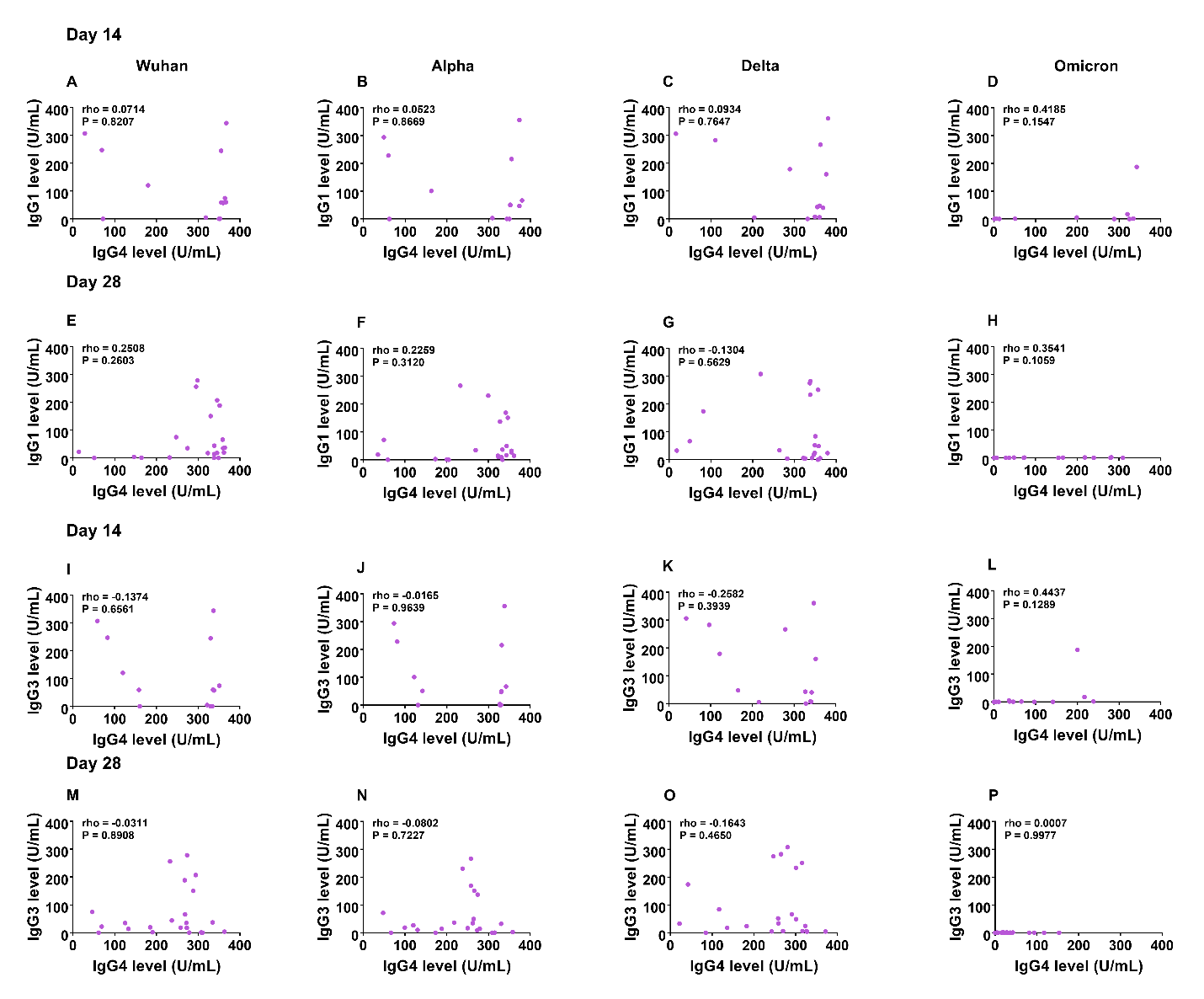
**

**Supplementary Figure S6**

Supplement: FIG S6 [file msphere.00465-22-s0006.docx]

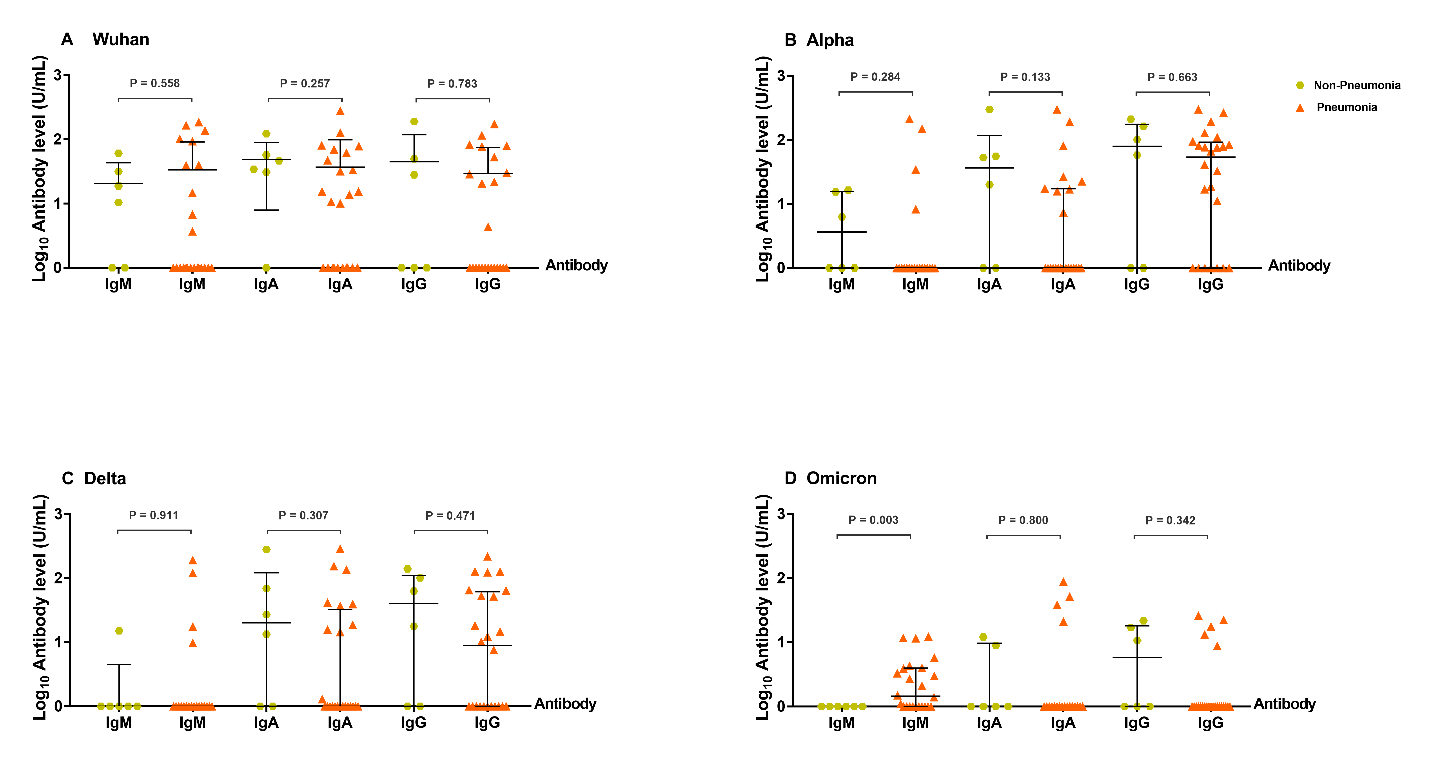


**Supplementary Figure S7**

Supplement: FIG S7 [file msphere.00465-22-s0007.docx]
