# Supplementary material for: Dynamics of Different Classes and Subclasses of Antibody Responses to Severe Acute Respiratory Syndrome Coronavirus 2 Variants after Coronavirus Disease 2019 and CoronaVac Vaccination in Thailand
Source: mSphere. 2023 Jan 23;8(1):e00465-22. doi: 10.1128/msphere.00465-22 (PMC9942573; doi:10.1128/msphere.00465-22)
Supplement: TABLE S1 [file msphere.00465-22-s0008.docx]

**Supplementary Table S1**

| Sample code | Type of vaccine | | | Vaccine interval between 1st and 2nd dose |
| --- | --- | --- | --- | --- |
|  | 1st dose | 2nd dose | 3rd dose |  |
| Covid-19-001 | NA | NA | NA | NA |
| Covid-19-002 | NA | NA | NA | NA |
| Covid-19-003 | CoronaVac | CoronaVac | NA | 3 weeks |
| Covid-19-004 | NA | NA | NA | NA |
| Covid-19-005 | NA | NA | NA | NA |
| Covid-19-006 | NA | NA | NA | NA |
| Covid-19-007 | NA | NA | NA | NA |
| Covid-19-008 | AZD1222 | NA | NA | NA |
| Covid-19-009 | AZD1222 | NA | NA | NA |
| Covid-19-010 | NA | NA | NA | NA |
| Covid-19-011 | AZD1222 | NA | NA | NA |
| Covid-19-012 | CoronaVac | CoronaVac | NA | 3 weeks |
| Covid-19-013 | CoronaVac | CoronaVac | NA | 3 weeks |
| Covid-19-014 | BBIBP-CorV | NA | NA | NA |
| Covid-19-015 | NA | NA | NA | NA |
| Covid-19-016 | CoronaVac | CoronaVac | NA | 3 weeks |
| Covid-19-017 | CoronaVac | CoronaVac | AZD1222 | 5 weeks |
| Covid-19-018 | NA | NA | NA | NA |
| Covid-19-019 | CoronaVac | CoronaVac | NA | 3 weeks |
| Covid-19-020 | CoronaVac | CoronaVac | NA | 3 weeks |
| Covid-19-021 | NA | NA | NA | NA |
| Covid-19-022 | AZD1222 | NA | NA | NA |
| Covid-19-023 | CoronaVac | CoronaVac | NA | 4 weeks |
| Covid-19-024 | AZD1222 | NA | NA | NA |
| Covid-19-025 | AZD1222 | NA | NA | NA |
| Covid-19-026 | NA | NA | NA | NA |
| Covid-19-027 | NA | NA | NA | NA |
| Covid-19-028 | AZD1222 | NA | NA | NA |
| Covid-19-029 | AZD1222 | NA | NA | NA |
| Covid-19-030 | AZD1222 | NA | NA | NA |
