# Supplementary material for: Dynamics of Different Classes and Subclasses of Antibody Responses to Severe Acute Respiratory Syndrome Coronavirus 2 Variants after Coronavirus Disease 2019 and CoronaVac Vaccination in Thailand
Source: mSphere. 2023 Jan 23;8(1):e00465-22. doi: 10.1128/msphere.00465-22 (PMC9942573; doi:10.1128/msphere.00465-22)
Supplement: TABLE S2 [file msphere.00465-22-s0009.docx]

**Supplementary Table S2**

| Antibody | Median (IQR) of antibodies against RBD of SARS-CoV-2 strains | | | | | | | | | | | |
| --- | --- | --- | --- | --- | --- | --- | --- | --- | --- | --- | --- | --- |
|  | Wuhan | | | Alpha (B.1.1.7) variant | | | Delta (B.1.617.2) variant | | | Omicron (B.1.1.529) variant | | |
|  | Day 0 | Day 14 | Day 28 | Day 0 | Day 14 | Day 28 | Day 0 | Day 14 | Day 28 | Day 0 | Day 14 | Day 28 |
| IgM | 1.9 (0-33.6) | 136.9 (15.7-231.0) | 25.2 (20.0-187.3) | 0 | 9.3 (0-99.9) | 21.5 (5.4-37.5) | 0 | 16.6 (0-51.4) | 33.8 (13.9-107.7) | 0.9 (0-2.8) | 2.1 (0-11.0) | 2.5 (0-7.0) |
| IgA | 14.6 (0-66.2) | 162.0 (89.2-248.0) | 73.8 (24.9-228.7) | 0 (0-30.3) | 225.4 (129.4-275.7) | 101 (15.8-248.4) | 0 (0-20.9) | 219.4 (84.9-267.0) | 125.7 (44.8-231.2) | 0 (0-2.2) | 117.7 (4.5-181.0) | 1.4 (0-97.8) |
| IgG | 28.4 (0-78.1) | 223.8 (54.5-274.0) | 123.1 (87.9-200.4) | 82.7 (14.1-137.5) | 208.4 (69.4-253.0) | 110.5 (66.0-178.5) | 35.6 (0-107.1) | 218.7 (38.4-273.9) | 139.6 (73.2-224.1) | 0.2 (0-17.2) | 221.9 (56.6-242.9) | 95.9 (39.6-191.7) |
| IgG1 | 4.1 (0-52.0) | 355.5 (210.7-365.2) | 345.4 (322.0-358.9) | 4.5 (0-48.0) | 351.8 (204.9-373.7) | 337.8 (318.2-349.0) | 6.1 (0-32.8) | 360.3 (221.2-372.3) | 347.6 (337.5-356.4) | 1.7 (0.9-2.7) | 288.4 (26.1-329.4) | 114.3 (29.6-249.8) |
| IgG2 | 13.5 (0-35.4) | 35.1 (13.7-98.6) | 31.3 (19.5-79.4) | 10.5 (1.2-26.0) | 24.5 (11.0-83.5) | 20.4 (8.9-33.4) | 20.9 (5.9-72.9) | 46.3 (9.1-120.6) | 21.8 (0-42.6) | 0 | 0 (0-10.1) | 0 |
| IgG3 | 20.8 (0-46.4) | 330.1 (120.6-337.6) | 262.8 (172.0-289.5) | 17.4 (0-53.6) | 330.6 (111.4-335.0) | 254.1 (173.7-268.0) | 22.5 (0-49.3) | 327.0 (131.3-344.7) | 272.9 (171.0-304.8) | 0.7 (0-4.8) | 96.7 (20.3-208.6) | 25.3 (15.9-84.7) |
| IgG4 | 49.9 (7.1-210.2) | 74.8 (58.4-277.5) | 40.7 (17.1-193.3) | 39.6 (13.1-190.3) | 66.9 (48.2-260.9) | 34.3 (13.5-155.7) | 42.6 (0.8-197.9) | 160.6 (41.6-294.4) | 59.2 (22.3-257.1) | 0 (0-0.3) | 1.3 (0.2-11.4) | 0 (0-1.1) |
