# Supplementary material for: Dynamics of Different Classes and Subclasses of Antibody Responses to Severe Acute Respiratory Syndrome Coronavirus 2 Variants after Coronavirus Disease 2019 and CoronaVac Vaccination in Thailand
Source: mSphere. 2023 Jan 23;8(1):e00465-22. doi: 10.1128/msphere.00465-22 (PMC9942573; doi:10.1128/msphere.00465-22)
Supplement: TABLE S3 [file msphere.00465-22-s0010.docx]

**Supplementary Table S3**

| Antibody | Median (IQR) of antibodies against RBD of SARS-CoV-2 strains | | | | | | | | | | | |
| --- | --- | --- | --- | --- | --- | --- | --- | --- | --- | --- | --- | --- |
|  | Wuhan | | | Alpha (B.1.1.7) variant | | | Delta (B.1.617.2) variant | | | Omicron (B.1.1.529) variant | | |
|  | Day 0 | Day 14 | Day 28 | Day 0 | Day 14 | Day 28 | Day 0 | Day 14 | Day 28 | Day 0 | Day 14 | Day 28 |
| IgM | 0 (0-55.5) | 93.4 (15.1-251.6) | 54.4 (29.5-154.5) | 0 (0-27.5) | 134.4 (62.1-232.1) | 118.2 (54.8-207.8) | 0 (0-15.7) | 196.5 (46.2-222.4) | 103.1 (36.1-149.2) | 0.9 (0-4.0) | 52.9 (3.8-94.6) | 5.8 (1.0-19.6) |
| IgA | 23.7 (0-43.3) | 254.8 (120.9-286.2) | 74.3 (41.5-258.5) | 3.7 (0-25.0) | 163.7 (41.3-290.7) | 206.0 (80.9-253.1) | 7.9 (0-119.5) | 275.2 (169.5-298.8) | 209.2 (64.2-267.7) | 0 (0-0.2) | 71.3 (12.3-158.9) | 2.6 (0-49.0) |
| IgG | 0 | 223.8 (150.3-244.9) | 182.8 (83.2-260.0) | 14.1 (0-64.4) | 183.6 (133.9-213.1) | 182.2 (93.6-258.0) | 0.2 (0-15.9) | 229.6 (165.0-258.0) | 166.9 (92.4-279.7) | 0 | 39.1 (4.6-71.3) | 32.3 (2.4-52.5) |
| IgG1 | 0 | 249.1 (98.9-341.9) | 239.4 (150.7-310.9) | 0 | 235.9 (87.3-335.0) | 202.9 (80.5-309.7) | 0 (0-0.7) | 319.2 (225.1-357.2) | 302.0 (127.3-325.1) | 0.9 (0.1-1.9) | 4.2 (1.4-11.6) | 2.8 (0.9-4.1) |
| IgG2 | 10.2 (3.1-14.4) | 13.9 (12.3-15.7) | 11.8 (6.6-27.7) | 8.8 (3.3-26.4) | 8.2 (6.4-11.4) | 8.2 (3.2-16.9) | 8.1 (3.5-13.8) | 12.9 (6.6-16.8) | 9.3 (0-18.6) | 0 | 0 (0-0.9) | 0 |
| IgG3 | 1.2 (0-7.7) | 241.1 (129.8-330.8) | 272.2 (99.2-299.9) | 1.5 (0-8.3) | 229.2 (124.2-329.3) | 267.8 (118.0-305.8) | 2.1 (0-10.7) | 277.5 (145.3-340.8) | 264.5 (92.5-326.9) | 0 | 24.4 (2.8-44.0) | 11.0 (0-27.3) |
| IgG4 | 17.1 (3.0-109.3) | 3.0 (0.7-91.9) | 10.7 (1.3-31.9) | 12.6 (3.6-99.1) | 2.0 (0.3-76.5) | 11.9 (1.1-30.4) | 17.2 (0.4-156.1) | 7.0 (5.3-136.0) | 5.8 (4.7-33.5) | 0.6 (0-2.7) | 0.7 (0.2-0.9) | 0 (0-0.4) |
